# Simultaneous atomic-resolution electron ptychography and *Z*-contrast imaging of light and heavy elements in complex nanostructures

**DOI:** 10.1038/ncomms12532

**Published:** 2016-08-26

**Authors:** H. Yang, R. N. Rutte, L. Jones, M. Simson, R. Sagawa, H. Ryll, M. Huth, T. J. Pennycook, M.L.H. Green, H. Soltau, Y. Kondo, B. G. Davis, P. D. Nellist

**Affiliations:** 1 Department of Materials, University of Oxford, Parks Road, OX1 3PH Oxford, UK; 2 Department of Chemistry, University of Oxford, 12 Mansfield Road, OX1 3TA Oxford, UK; 3 PNDetector GmbH, Sckellstrasse 3, 81667 München, Germany; 4 JEOL Ltd 3-1-2 Musashino Akishima, Tokyo 196-8558, Japan; 5 PNSensor GmbH, Otto-Hahn-Ring 6, 81739 München, Germany; 6 Department of Physics, University of Vienna, Boltzmanngasse 5, 1090 Vienna, Austria

## Abstract

The aberration-corrected scanning transmission electron microscope (STEM) has emerged as a key tool for atomic resolution characterization of materials, allowing the use of imaging modes such as *Z*-contrast and spectroscopic mapping. The STEM has not been regarded as optimal for the phase-contrast imaging necessary for efficient imaging of light materials. Here, recent developments in fast electron detectors and data processing capability is shown to enable electron ptychography, to extend the capability of the STEM by allowing quantitative phase images to be formed simultaneously with incoherent signals. We demonstrate this capability as a practical tool for imaging complex structures containing light and heavy elements, and use it to solve the structure of a beam-sensitive carbon nanostructure. The contrast of the phase image contrast is maximized through the post-acquisition correction of lens aberrations. The compensation of defocus aberrations is also used for the measurement of three-dimensional sample information through post-acquisition optical sectioning.

## Abstract (web summary)

The use of ptychography with electrons has been limited. Here, Yang *et al*. demonstrate that the combination of Z-contrast and phase imaging reveals the structure of complex nanomaterials. This practical tool can be used to solve the structure of a beam-sensitive carbon nanostructure at atomic-resolution.

## Graphical abstract

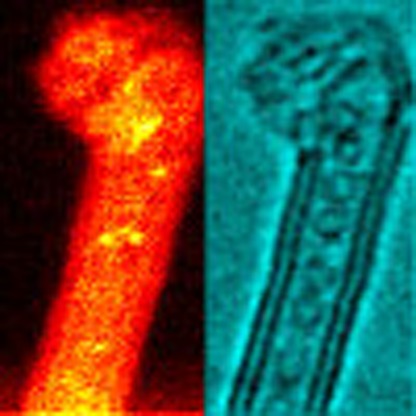

Forming an atomic-resolution image in the electron microscope that provides dose-efficient, high-contrast imaging of light elements, while also providing strong compositional contrast, has been a long-standing challenge. For light materials, such as graphene and biological structures, high-energy electrons are predominantly only scattered once in the sample. Such single scattering conditions can be described as a weak-phase object (WPO)^1^, where only a small phase shift is introduced to the electron wave. Phase-contrast imaging using electrons has been shown to provide the highest dose efficiency for imaging WPOs, where radiation damage is a limiting factor^2^, compared with inelastic electron scattering, as well as X-ray and neutron scattering. Electric and magnetic fields also lead to a phase shift of the electron beam. Phase-contrast imaging therefore plays an important role in the imaging of biological structures, light materials such as graphene and the detection of electric and magnetic fields. It is generally performed in the conventional transmission electron microscope (CTEM) using either deliberately injected lens aberrations or a phase plate^3^, to form image contrast of a WPO, or using off-axis holography^4^,^5^, which requires a separate reference beam deflected using an electron bi-prism.

Providing a strong compositional sensitivity to element type, however, requires the detection of relatively high-angle scattering and this is performed using annular dark-field (ADF) imaging in the scanning TEM (STEM), as shown in Fig. 1, which forms an image with atomic-number contrast (*Z*-contrast). A beam of electrons is focused to form a probe of atomic dimensions that is raster scanned across the sample and the ADF detector collects the intensity of the high-angle scattering that is then plotted as a function of probe position to form an image.

The *Z*-contrast technique has developed into a key approach for atomic-resolution imaging, in particular as the development of hardware correctors for the inherent spherical aberration of the objective lens^6^,^7^. It is an incoherent imaging technique, which means that the optimal conditions for imaging are the smallest, most intense illuminating probe possible^8^. This is achieved by tuning the aberration corrector for the smallest possible aberrations. Phase-contrast imaging, however, is a coherent imaging mode and requires aberrations, such as a combination of spherical aberration and defocus, to form an effective phase plate. Such conditions are referred to as ‘Scherzer conditions'^9^. Alternative approaches that also make use of aberrations include negative spherical aberration conditions^10^ or the use of a focal series^11^ in TEM. The optimal conditions for incoherent *Z*-contrast imaging are therefore different from those for coherent phase contrast, thus preventing their simultaneous optimal implementation. Furthermore, the principle of reciprocity^12^ shows that the formation of a conventional phase-contrast image in STEM requires the use of a small axial bright-field (BF) detector, which collects only a small fraction of the transmitted electrons and is therefore dose inefficient.

Here we make use of electron ptychography to enable an efficient, quantitative phase imaging in STEM under optical conditions that are optimized for incoherent imaging so that the phase information is recorded simultaneously with the *Z*-contrast image, allowing a full analysis. Although ptychography has become well established for imaging with light^13^ and X-rays^14^,^15^,^16^, its use with electrons has been more limited. The principle of ptychography is that a camera is used to record the intensity in the STEM detector plane as a function of probe position, resulting in a four-dimensional (4D) data set (two STEM probe coordinates and two coordinates within the detector plane), which can be used to reconstruct a phase image. Initial experimental demonstrations of electron ptychography were limited by the camera speed, microscope stability and the availability of sufficient computational processing power, and only very small images (typically 32 × 32 pixels) could be reconstructed^17^. More recent demonstrations have used highly defocused probes that illuminate a wider region of the sample to provide a larger field of view, while still limiting the number of probe positions and camera frames^18^,^19^,^20^. Such defocused probes are incompatible with the conditions required for incoherent imaging and so cannot be used simultaneously with *Z*-contrast imaging. The subsequent formation of a *Z*-contrast image would require refocusing of the electron probe, which requires further electron dose on the sample and loses the ability to exactly register the phase and *Z*-contrast information. A first proof-of-principle attempt to form simultaneous ADF and phase-contrast images^21^ was also limited in field of view by the camera speed. Differential phase contrast (DPC) images have been formed at atomic resolution in the STEM and used to detect ferroelectric polarization^22^, and the importance of adding phase-sensitive imaging as a STEM technique discussed^23^, but the differential approach did not offer quantitative image phase measurement and does not offer the possibility of post-acquisition aberration correction demonstrated here. Ptychography has been shown to enable phase extraction using signals from segmented detectors including tri-sectors^24^ and recently at atomic resolution using segments synthesized from pixelated data^25^. Large detector pixels may lead to a non-isotropic transfer of phase information with reduced phase-contrast transfer efficiencies^26^.

Here we show that new capabilities that have been enabled by recent developments in high-speed detector technology (described further in the Methods section) that provide frame speeds of 1 kHz or greater allowing image acquisition at scanning speeds much closer to those typically used in STEM. We show that the combination of *Z*-contrast and phase imaging reveals the structure of a complex nanomaterial that could not be determined using previous methods. Ptychography also allows for further post-acquisition detection and correction of residual lens aberrations, which optimizes the contrast of the phase image. We also show that ptychographic reconstruction can be used to access three-dimensional (3D) information through its intrinsic optical-sectioning effect.

## Results

### Data acquisition and processing

**Figure 1:**
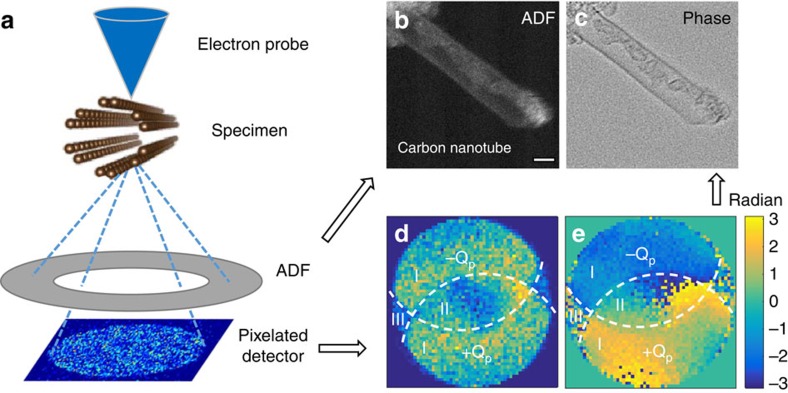
Simultaneous atomic resolution incoherent and coherent imaging. (**a**) An ADF detector collects the dark-field signal to form (**b**) an incoherent *Z*-contrast image. (**c**) The simultaneous phase image is reconstructed using ptychography. Simultaneously, a fast pixelated detector records the coherent BF diffraction pattern at every probe position forming a 4D data set. Taking the Fourier transform of the 4D data set with respect to probe position results in a complex 4D matrix *G*(**K**_**f**_, **Q**_**p**_), which carries the phase information of the interference between diffracted and undiffracted beams. (**d**,**e**) An example of the modulus and phase of the complex matrix *G*(**K**_**f**_, **Q**_**p**_) at a single spatial frequency **Q**_**p**_ where two diffracted beams +**Q**_**p**_ and −**Q**_**p**_ (indicated by dashed lines) overlap with the undiffracted direct beam. (**d**) The areas labelled as area I are double-overlap regions where one diffracted beam interferes with the direct beam and area II is the triple-overlap region where both diffracted beams and the direct beam interfere. Area III has no interfering beams and is only noise and is therefore not used in the ptychography reconstruction. By analysing the phase information for all spatial frequencies in the image, (**c**) the phase image can be reconstructed. Scale bar, 1 nm.

Figure 1a shows a schematic of the optical configuration for the experiment presented here, with single frames of the experimental diffraction patterns shown in Supplementary Fig. 1. Further experimental details are described in the Methods section. As a highly convergent beam is used in STEM, the diffracted beams observed in the far field broaden into discs. The phase information is found where these discs overlap. Using a fast pixelated STEM detector, diffraction patterns consisting of primarily the BF disc (direct beam) were recorded at every probe position during a raster scan, thereby forming a 4D data set. The dark-field signal is simultaneously collected using an ADF detector, to form an incoherent *Z*-contrast image, as shown in Fig. 1b. In the detector plane, the recorded electron micro-diffraction patterns as a function of scattering vector **K**_**f**_ and probe position **R**_**0**_, can be described as the Fourier transform of product of the probe *P*(**R**−**R**_**0**_) and the specimen transmission function 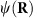,


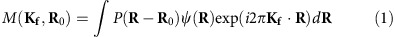


The intensity of the diffraction pattern 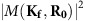 is recorded but the phase is lost. Various ptychography approaches in electron and X-ray microscopy have been developed. Here we used the Wigner distribution deconvolution (WDD) approach described by Rodenburg and Bates^27^, and demonstrated experimentally using X-rays^28^. WDD is non-iterative and therefore fast, especially under the conditions presented here where 256 × 256 image pixels results in 65,536 individual diffraction patterns to be processed. Iterative approaches^14^,^18^,^29^,^30^,^31^ and direct inversion methods^19^ have also been developed, but as yet they have not been demonstrated on the type of atomic-scale focused probe data used here, to enable the simultaneous incoherent imaging. The feasibility of using iterative methods on this type of data will be explored in further work.

WDD is performed by taking the forward Fourier transform of 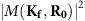 in equation (1) with respect to the probe positions **R**_**0**_, leading to a complex 4D matrix denoted as *G*(**K**_**f**_, **Q**_**p**_),^27^


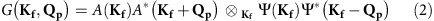


where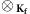 denotes a convolution with respect to the scattering vector **K**_**f**_ and the two terms 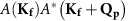 and 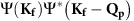 are functions of the probe forming aperture, *A*(**K**_**f**_), and the specimen transmission function, 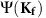, respectively. They have been shown to follow the mathematical definition of the Fourier transform of a Wigner distribution function^27^. *G*(**K**_**f**_, **Q**_**p**_) contains information about the beam interferences at disc overlaps^26^ (see Supplementary Methods), with an example shown in Fig. 1d,e. Such interference at disc overlaps contain both phase information of the specimen under illumination and the phase information due to residual lens aberrations, which will be discussed later (see Supplementary Fig. 2 and Supplementary Table 1). In the absence of lens aberrations, the two double-overlap regions (labelled as area I in Fig. 1d,e) should be uniform in phase, although small residual aberrations cause small variations in phase in these regions. Equation (2) shows that *G*(**K**_**F**_, **Q**_**p**_) can be expressed as the result of a convolution between a function representing disc overlaps and a function derived from the specimen. The disc overlap function can be deconvolved from equation (2) and the object transmission function (Fig. 1c) can be reconstructed from 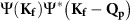 by setting **K**_**f**_ to zero. The WDD approach does not rely on the WPO approximation, but does require the transmission function to be a multiplicative object acting on the incident wave. As such, it is not strictly applicable to dynamical electron scattering, but none of the examples presented here would be regarded as leading to dynamical scattering conditions. The deconvolution step in the WDD enables residual lens aberrations, which affect the phase of the aperture function, *A*(**K**_**f**_), to be corrected from the phase image post-processing. The aberration correction enabled by WDD further allows 3D optical sectioning to be performed using one 4D data set at a fixed defocus without the need to run a focal-series experiment.

### Simultaneous *Z*-contrast and phase of a complex nanostructure

**Figure 2:**
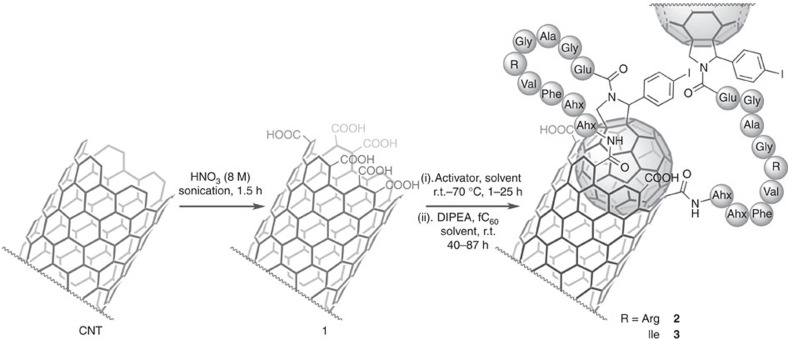
Scheme of the intended hybrid synthesis. CNTs were oxidized to introduce carboxylic acid groups for functionalization. CNT-COOH **1** then underwent carboxyl preactivation and subsequent intended reaction with amine-bearing modified C_60_ peptido-fullerene **fC**_**60**_, to give the target tethered hybrids **2** and **3**. Ahx denotes aminohexanoyl residue. Fo all other amino acid residues, standard 3-letter amino acid codes are used.

The combined ptychography and *Z*-contrast approach was tested on complex carbon nanotube (CNT) conjugates, which have exciting potential biological or medicinal applications, yet remain highly challenging to characterize. As a representative analytical substrate, candidate drug-delivery materials **2** and **3** (see Supplementary Methods) were chosen. As shown in Fig. 2, the intended design of this system aimed to create a peptidic covalently attached ‘tether' between a single-walled CNT and a limited number of carbon fullerene (C_60_) molecules. In this work, conventional analytical data (including infrared, Raman, X-ray photoemission spectroscopy and thermogravimetric analysis (TGA)) and the CTEM results suggested potentially consistent results with the intended synthesis (see Supplementary Figs 3–11); however, the absolute evidence for attachment and purity remained ambiguous.

**Figure 3:**
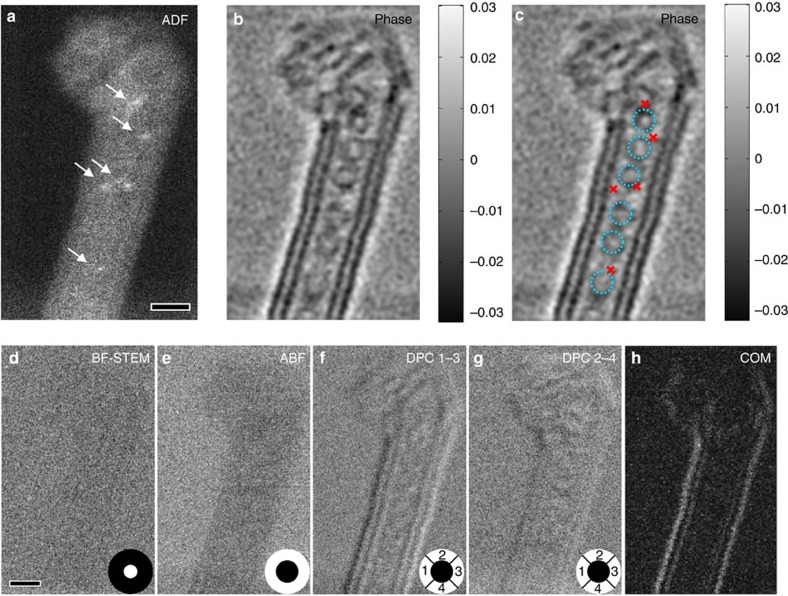
Simultaneous *Z*-contrast and phase images of a double-wall CNT peapod. (**a**) Incoherent *Z*-contrast ADF image clearly shows the locations of the single iodine atoms indicated by the arrows. (**b**) The reconstructed phase image shows the presence of fullerenes inside the CNT. (**c**) Annotated phase image with the fullerenes labelled using dotted circles and iodine atoms labelled using cross marks based on their locations in the ADF image. It is clear that the iodine atoms are located close to but outside the fullerenes. For comparison, conventional phase-contrast images including BF, ABF, DPC and the DPC using the centre of mass (COM) approach were synthesized from the data and shown in **d**–**h**, respectively. The detector area of each imaging method is shown in white colour in **d**–**g**. The experiment was performed at an electron probe current of ∼2.8 pA, pixel dwell time of 0.25 ms and a dose of ∼1.3 × 10^4^ e^−^ Å^−2^. Scale bar, 1 nm; the grey scale of the phase in **b** is in unit of radians.

Vitally, this ambiguity was resolved by the simultaneous *Z*-contrast and phase imaging demonstrated here. As shown in Fig. 3, the *Z*-contrast image reveals the position of the iodine atoms. These are not visible in the simultaneously acquired phase image because of its low atomic number sensitivity. Similarly, the carbon nanostructures are not clearly visible in the *Z*-contrast image. It is the ability created here to combine these imaging modes that allows the structure to be fully determined. The phase image clearly reveals a CNT containing deeply engulfed fullerene molecules that have entered the tubes to a depth that is inconsistent with the length of the shorter peptide tether. These observed ‘peapod' structures thus uniquely revealed that not all of the intended attachment links between fullerene and CNT had been successful, and hence vitally informed redesign and function of the hybrid system.

Previous atomic resolution imaging of CNTs containing fullerenes has been achieved using phase contrast in the CTEM^32^,^33^ and heavy dopant atoms in these systems revealed using *Z*-contrast imaging in STEM^34^,^35^,^36^, sometimes combined with BF imaging or electron energy loss spectroscopy. In the case of STEM, imaging of a WPO using BF imaging in STEM relies on deliberate defocus aberrations to form necessary contrast^37^, which reduce the *Z*-contrast image resolution and contrast. To demonstrate this, other existing phase-sensitive imaging modes, including the conventional BF, annular BF^38^,^39^ (ABF), DPC^22^ and ‘first moment' DPC imaging^40^,^41^ were also synthesized from the same 4D data set (in Fig. 3d–h); however, none of these modes are able to image the ‘peapod' structure clearly at the electron dose used because of their worse signal-to-noise under conditions of near-zero aberrations. As expected, BF and ABF show the worst contrast of all imaging methods, because, similar to all symmetric detectors, they offer no weak-phase contrast under conditions of zero aberrations^26^. Furthermore, the WDD method makes use of only the regions of the detector containing signal (for example, only regions I and II in Fig. 1d) and hence rejects noise more effectively than these other modes, which use the entire BF disc.

### Measurement and correction of residual lens aberrations

Equation (2) shows that WDD allows residual lens aberrations to be deconvolved from the 4D data set, leading towards aberration-free phase imaging. Here we demonstrate that the redundancy of information inside the 4D data also allows the illuminating probe function to be measured directly. This capability has been demonstrated with iterative methods for highly defocused data^14^,^29^ and here we implement a direct measurement for in-focus data of low- and high-order aberrations by solving a set of linear equations. Specifically, the phase of *G*(**K**_**f**_, **Q**_**p**_) in the disc overlap regions can be mathematically described as a linear function of the lens aberration coefficients under the WPO approximation (see Supplementary Methods), and in this work we use this phase to measure the aberrations through a deterministic matrix inversion to solve such a set of linear equations (see Supplementary Fig. 2 and Supplementary Table 1). This aberration measurement method is a fast direct method that can be applied to both crystalline and non-crystalline specimens.

**Figure 4:**
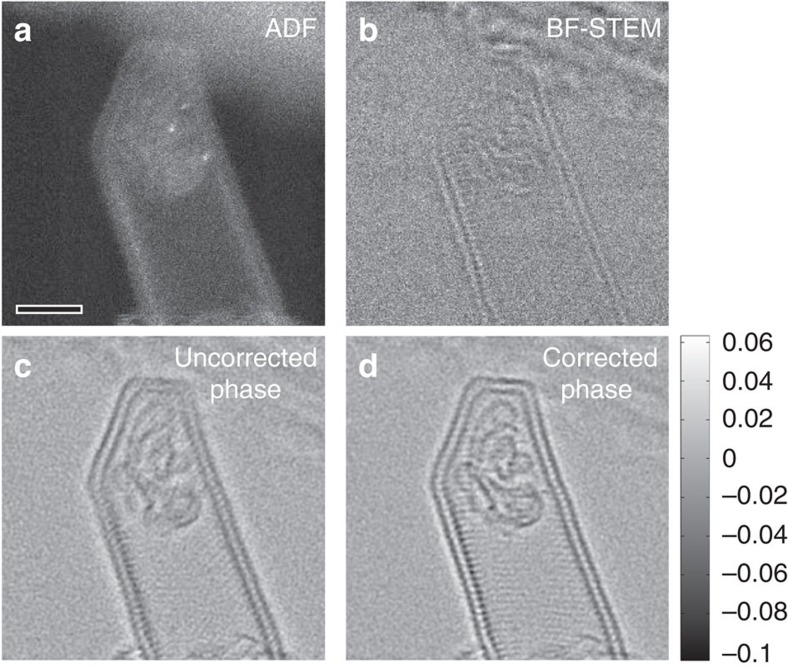
Imaging a double-wall CNT at atomic resolution. (**a**) The ADF image shows the presence of single iodine dopants, whereas (**b**) the simultaneous BF image is rather noisy and shows little contrast of the CNT. (**c**,**d**) The phase image before and after correcting residual aberrations, respectively. This comparison shows the improved visibility of the CNT atomic fringes and the damaged carbonaceous structure inside the CNT due to aberration correction. Scale bar, 2 nm.

Figure 4 shows the result of applying the residual aberration correction to the phase image of another double-walled CNT structure recorded at ∼2.7 × 10^4^ e^−^ Å^−2^ dose, which is twice that used for Fig. 3. Electron irradiation has destroyed the internal fullerene structures. Detection and subsequent deconvolution of a small residual aberration of 3.5 nm defocus and 2 nm twofold astigmatism allows the visibility of the lattice in the walls of the CNT to be significantly improved (Fig. 4d). Fringes from the lattice of the CNT are visible primarily along only one direction in the uncorrected phase (in Fig. 4c), whereas the corrected phase (Fig. 4d) shows crossed fringes along with a visibly improved contrast. This aberration-corrected image demonstrates the quality of phase images that can now be achieved in a STEM using ptychography. The improved quality of phase images enabled by post-processing aberration correction can be quite beneficial for imaging beam-sensitive materials. Relaxing the requirement to manually adjust the probe defocus and astigmatism while imaging the sample, leads to a decrease in the electron dose and damage.

### WDD optical sectioning from a 4D data set of single defocus

**Figure 5:**
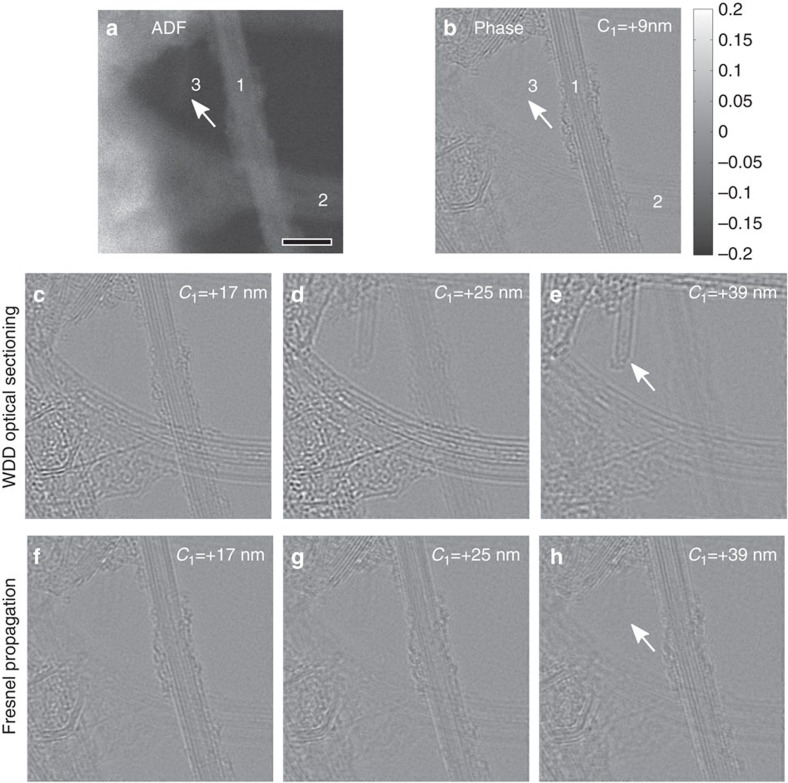
WDD 3D optical sectioning of crossed CNTs of different heights. (**a**) The simultaneous ADF image shows that none of the tubes is exactly in focus in the experimental data. By deconvolving a series of defocus aberrations (C_1_) from the 4D data set, a set of reconstructed phase images are obtained. The best phase image contrast of the two crossed nanotubes (labelled as 1 and 2 in **a**) are found to be at defocus (**b**) +9 nm and (**d**) +25 nm, respectively, with ‘+' being over-focus relative to the experimental focal plane, and the mid-plane between the two optimal focus is shown in **c** as a comparison. (**e**) At defocus +39 nm, a small CNT filled with fullerene (indicated by the arrow) becomes visible. WDD optical sectioning offers a depth sensitivity that is not equivalent to the simple Fresnel wave propagation of the reconstructed ‘exit wave' and this is evidenced from **f**,**g**,**h** obtained by propagating the ptychographically reconstructed complex object function in **b** to the corresponding heights in **c**–**e**. The white arrow points to the small CNT, which becomes visible using WDD optical sectioning in **e**, but it remains invisible after applying Fresnel propagation in **h**, in comparison. Scale bar, 5 nm.

A further advantage of the ptychographic 4D data set is that it contains 3D structure information of the object, as previously demonstrated for light microscopy^42^,^43^. An object that is out of focus still has its information encoded in the 4D data set, but the disc overlap described by the 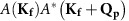 term in equation (2) will show a characteristic phase variation. We find that if the WDD process is performed using a defocus value that does not correspond to the depth of the object, the phase variations in the disc overlap region will not match the deconvolution kernel and will act to reduce the contribution of the object to the reconstructed phase image. Only when the correct defocus is used will the object fully appear and thus an ‘optical sectioning' effect can be used to gain 3D structure information (see Supplementary Methods), even though the data has been recorded using only one experimental defocus with only one scan of the electron beam. Figure 5 shows an example of crossed CNTs located at different heights along the electron beam direction. A series of reconstructed phases were obtained from a single data set by deconvolving a series of defocus (C_1_) values from the recorded 4D data set, whereupon the various CNTs located at different depths along the electron beam direction become most visible at different defocus (C_1_) values used in WDD. For example, a small nanotube (indicated by arrows in Fig. 5) that shows no contrast in Fig. 5b,c becomes gradually visible in Fig. 5d,e, as the deconvolution kernel focuses at different depths (also see Supplementary Movie 1). The relative heights of the CNTs can be easily identified by analysing the contrast of the phase images.

It should be emphasized that this is a true optical sectioning effect enabled by WDD under the kinematical approximation and is not equivalent to a Fresnel propagation of the ‘exit wave' as in high-resolution TEM (HRTEM)^44^,^45^. As can be seen in Fig. 5f–h, Fresnel propagation of the complex transmission function retrieved using a ptychographic reconstruction using a defocus value giving the highest contrast for one CNT does not recover the phase information of the other CNTs located at different depths. Using WDD, by deconvolving the defocus aberration corresponding to a given depth, the deconvolution process acts to enhance the transfer of information at the depth selected by the deconvolution kernel and suppresses the transfer of out-of-focus phase information (see Supplementary Methods); therefore, each phase image resulted from WDD optical sectioning provides a unique identification of the 3D structure at its corresponding depth. We further demonstrate that WDD optical sectioning leads to a depth resolution and a bound to the 3D transfer function (see Supplementary Methods and Supplementary Figs 12 and 13), which corresponds to that for ADF STEM optical sectioning^46^. The fact that 3D optical sectioning can be achieved by scanning the sample only once at a fixed defocus without the need to run a focal series imaging significantly reduces the electron dose compared with the conventional STEM ADF optical sectioning methods^47^,^48^. The ability to measure and correct high-order aberrations with post-processing enables further opening up of the probe forming aperture to reduce the depth of field of the microscope^49^ for 3D phase imaging in future studies.

## Discussion

We have shown that developments in camera and information handling technologies have allowed the use of ptychography to form phase images in the STEM under the zero-aberration conditions required for optimal simultaneous incoherent *Z*-contrast imaging. It is also shown that, for this type of focused-probe data set, the direct WDD approach provides high-quality reconstructions and allows correction of small residual aberrations. The ability to deconvolve aberrations leads to the ability to optically section samples with a nanometre-scale depth resolution.

The ability to form simultaneous phase images and incoherent images has further potential, for example, simultaneous formation of energy-dispersive X-ray maps, or other spectroscopic and secondary electron imaging^50^, with the phase image. Recording electron energy-loss spectra would, of course, require retraction of the fast camera, but phase and electron energy loss spectroscopy maps could be formed consecutively without any adjustment to the microscope electron optics required.

As the majority of the scattered electrons from the weak-scattering nanomaterials used here were detected, we would expect the dose requirements of the approach presented here to be similar to that for conventional HRTEM, but a detailed analysis is beyond the scope of this study. The ability to post-correct residual aberrations to improve contrast is shared with focal series exit-wave reconstruction in HRTEM^51^ but for low-dose imaging the re-registration of the focal series of images can be challenging, whereas no re-registration is required for ptychography.

Finally, we emphasise that 3D information is inherent in the data from a single scan and so optical sectioning can be performed post acquisition without the need for acquiring an experimental focal series of data.

## Methods

### pnCCD fast pixelated detector

The experiments were performed using the pnCCD (S)TEM camera, a direct electron, radiation hard pixelated detector from PNDetector^52^,^53^, mounted on the JEOL ARM200-CF aberration-corrected microscope with a 80 keV primary beam energy and 22 mrad convergence angle. This camera delivers full frame images of 264 × 264 pixels at a readout speed of 1,150 frames per second (fps) routinely and up to 20,000 fps through binning/windowing. The BF diffraction signal was set to cover almost the entire detector area by adjusting the camera length and the dark-field signal was collected by the ADF detector. This speed allows a typical 4D ptychographic data set, consisting of a 66 × 264 pixel image (fourfold binning) of the diffraction pattern acquired at each probe position in a 256 × 256 point grid, to be obtained in around 16 s. The data presented here were acquired in fourfold binning (4,000 fps) and twofold binning (2,000 fps) for the CNTs and the graphene, respectively. All data were taken in high charge handling mode^54^.

### Simultaneous synthetic images

Synthetic BF, ABF and DPC can also be obtained from the recorded diffraction patterns, with the full flexibility to adjust the collection angles and precise cantering of the ABF detector, as well as the partition direction of DPC segments. Because of the focused probe being used, *Z*-contrast images can be recorded simultaneously using an ADF detector.

### Wigner distribution deconvolution

The detailed procedure of WDD follows the previous literature by Rodenburg and Bates^27^. The probe function is obtained by measuring the aberration measurement algorithm described in the Supplementary Methods. The only difference of this work compared with the previous work^27^ is that a procedure called ‘stepping out' was implemented into the WDD algorithm in the previous work, aiming for improving the resolution of the reconstructed phase, whereas in this work, ‘stepping out' is not implemented, because the primary focus of WDD is for phase-sensitive imaging of weakly scattering objects instead of improving resolution. For the weakly scattering carbon nanostructures under study, ‘stepping out', relying as it does on the so-called nonlinear interference, is not available.

### Data availability

The numerical data shown in Figs 3, 4, 5 are available in TIFF format and the entire raw 4D data are all available. Links send on request to http://peter.nellist@materials.ox.ac.uk.

## Additional information

**How to cite this article:** Yang, H. *et al*. Simultaneous atomic-resolution electron ptychography and *Z*-contrast imaging of light and heavy elements in complex nanostructures. *Nat. Commun.* 7:12532 doi: 10.1038/ncomms12532 (2016).

## Supplementary Material

Supplementary Information Supplementary Figures 1-13, Supplementary Table 1, Supplementary Methods and Supplementary References.

Supplementary Movie 1 Optical sectioning using Wigner distribution deconvolution revealing the depth sensitivity to carbon nanotubes located at different heights along the e-beam direction.

## References

[b1] Cowley J. M. Diffraction Physics Elsevier (1995).

[b2] Henderson R. The potential and limitations of neutrons, electrons and X-rays for atomic-resolution microscopy of unstained biological molecules. Q. Rev. Biophys. 28, 171–193 (1995). 7568675 10.1017/s003358350000305x

[b3] Nagayama K. & Danev R. Phase-plate electron microscopy: a novel imaging tool to reveal close-to-life nano-structures. Biophys. Rev. 1, 37–42 (2009). 20585379 10.1007/s12551-008-0006-z PMC2883085

[b4] Lichte H., Reibold M., Brand K. & Lehmann M. Ferroelectric electron holography. Ultramicroscopy 93, 199–212 (2002). 12492231 10.1016/s0304-3991(02)00277-2

[b5] Linck M., Freitag B., Kujawa S., Lehmann M. & Niermann T. State of the art in atomic resolution off-axis electron holography. Ultramicroscopy 116, 13–23 (2012).

[b6] Haider M. . A spherical-aberration-corrected 200 kV transmission electron microscope. Ultramicroscopy 75, 53–60 (1998).

[b7] Krivanek O. L., Dellby N. & Lupini A. R. Towards sub-Å electron beams. Ultramicroscopy 78, 1–11 (1999).

[b8] Nellist P. D. & Pennycook S. J. The principles and interpretation of annular dark-field Z-contrast imaging. Adv. Imaging Electron Phys. 113, 147–203 (2000).

[b9] Scherzer O. The theoretical resolution limit of the electron microscope. J. Appl. Phys. 20, 20–29 (1949).

[b10] Jia C.-L., Lentzen M. & Urban K. High-resolution transmission electron microscopy using negative spherical aberration. Microsc. Microanal. 10, 174–184 (2004). 15306044 10.1017/S1431927604040425

[b11] Saxton W. Accurate atom positions from focal and tilted beam series of high resolution electron micrographs. Scanning Microsc. 2, 213–224 (1988).

[b12] Cowley J. M. Image contrast in a transmission scanning electron microscope. Appl. Phys. Lett. 15, 58–59 (1969).

[b13] Maiden A. M., Rodenburg J. M. & Humphry M. J. Optical ptychography: a practical implementation with useful resolution. Opt. Lett. 35, 2585–2587 (2010). 20680066 10.1364/OL.35.002585

[b14] Thibault P. . High-resolution scanning X-ray diffraction microscopy. Science 321, 379–382 (2008). 18635796 10.1126/science.1158573

[b15] Maiden A. M., Morrison G. R., Kaulich B., Gianoncelli A. & Rodenburg J. M. Soft X-ray spectromicroscopy using ptychography with randomly phased illumination. Nat. Commun. 4, 1669 (2013). 23575673 10.1038/ncomms2640

[b16] Dierolf M. . Ptychographic X-ray computed tomography at the nanoscale. Nature 467, 436–439 (2010). 20864997 10.1038/nature09419

[b17] Rodenburg J. M., McCallum B. C. & Nellist P. D. Experimental tests on double-resolution coherent imaging via STEM. Ultramicroscopy 48, 304–314 (1993).

[b18] Humphry M. J., Kraus B., Hurst A. C., Maiden A. M. & Rodenburg J. M. Ptychographic electron microscopy using high-angle dark-field scattering for sub-nanometre resolution imaging. Nat. Commun. 3, 730 (2012). 22395621 10.1038/ncomms1733 PMC3316878

[b19] D'Alfonso A. J. . Deterministic electron ptychography at atomic resolution. Phys. Rev. B 89, 064101 (2014).

[b20] Putkunz C. T. . Atom-scale ptychographic electron diffractive imaging of boron nitride cones. Phys. Rev. Lett. 108, 073901 (2012). 22401205 10.1103/PhysRevLett.108.073901

[b21] Pennycook T. J. . Efficient phase contrast imaging in STEM using a pixelated detector. Part 1: experimental demonstration at atomic resolution. Ultramicroscopy 151, 160–167 (2015). 25458189 10.1016/j.ultramic.2014.09.013

[b22] Shibata N. . Differential phase-contrast microscopy at atomic resolution. Nat. Phys. 8, 611–615 (2012).

[b23] Nellist P. D. Electron microscopy: atomic resolution comes into phase. Nat. Phys. 8, 586–587 (2012).

[b24] McCallum B. C., Landauer M. N. & Rodenburg J. M. Complex image reconstruction of weak specimens from a three-sector detector in the STEM. Optik 101, 53–62 (1995).

[b25] Brown H. G. . Structure retrieval with fast electrons using segmented detectors. Phys. Rev. B 93, 134116 (2016).

[b26] Yang H., Pennycook T. J. & Nellist P. D. Efficient phase contrast imaging in STEM using a pixelated detector. Part II: optimisation of imaging conditions. Ultramicroscopy 151, 232–239 (2015). 25481091 10.1016/j.ultramic.2014.10.013

[b27] Rodenburg J. M. & Bates R. H. T. The theory of super-resolution electron microscopy via Wigner-distribution deconvolution. Philos. Trans. R. Soc. A Math. Phys. Eng. Sci. 339, 521–553 (1992).

[b28] Chapman H. N. Phase-retrieval X-ray microscopy by Wigner-distribution deconvolution. Ultramicroscopy 66, 153–172 (1996).

[b29] Maiden A. M. & Rodenburg J. M. An improved ptychographical phase retrieval algorithm for diffractive imaging. Ultramicroscopy 109, 1256–1262 (2009). 19541420 10.1016/j.ultramic.2009.05.012

[b30] Hüe F., Rodenburg J. M., Maiden A. M., Sweeney F. & Midgley P. A. Wave-front phase retrieval in transmission electron microscopy via ptychography. Phys. Rev. B 82, 121415 (2010).

[b31] Maiden A. M., Sarahan M. C., Stagg M. D., Schramm S. M. & Humphry M. J. Quantitative electron phase imaging with high sensitivity and an unlimited field of view. Sci. Rep. 5, 14690 (2015). 26423558 10.1038/srep14690 PMC4589788

[b32] Hirahara K. . One-dimensional metallofullerene crystal generated inside single-walled carbon nanotubes. Phys. Rev. Lett. 85, 5384–5387 (2000). 11136002 10.1103/PhysRevLett.85.5384

[b33] Koshino M. . Analysis of the reactivity and selectivity of fullerene dimerization reactions at the atomic level. Nat. Chem. 2, 117–124 (2010). 21124402 10.1038/nchem.482

[b34] Colliex C. . Capturing the signature of single atoms with the tiny probe of a STEM. Ultramicroscopy 123, 80–89 (2012). 22626784 10.1016/j.ultramic.2012.04.003

[b35] Suenaga K. . Visualizing and identifying single atoms using electron energy-loss spectroscopy with low accelerating voltage. Nat. Chem. 1, 415–418 (2009). 21378897 10.1038/nchem.282

[b36] Arenal R. . Atomic configuration of nitrogen-doped single-walled carbon nanotubes. Nano Lett. 14, 5509–5516 (2014). 25157857 10.1021/nl501645g

[b37] Nicholls R. J. . Direct imaging and chemical identification of the encapsulated metal atoms in bimetallic endofullerene peapods. ACS Nano 4, 3943–3948 (2010). 20557070 10.1021/nn100823e

[b38] Ishikawa R. . Direct imaging of hydrogen-atom columns in a crystal by annular bright-field electron microscopy. Nat. Mater. 10, 278–281 (2011). 21317899 10.1038/nmat2957

[b39] Findlay S. D. . Robust atomic resolution imaging of light elements using scanning transmission electron microscopy. Appl. Phys. Lett. 95, 10–13 (2009).

[b40] Waddell E. & Chapman J. Linear imaging of strong phase objects using asymmetrical detectors in STEM. Optik 54, 83–96 (1979).

[b41] Müller K. . Atomic electric fields revealed by a quantum mechanical approach to electron picodiffraction. Nat. Commun. 5, 5653 (2014). 25501385 10.1038/ncomms6653 PMC4275586

[b42] Maiden A. M., Humphry M. J. & Rodenburg J. M. Ptychographic transmission microscopy in three dimensions using a multi-slice approach. J. Opt. Soc. Am. A 29, 1606–1614 (2012). 10.1364/JOSAA.29.001606 23201876

[b43] Li P., Batey D. J., Edo T. B. & Rodenburg J. M. Separation of three-dimensional scattering effects in tilt-series Fourier ptychography. Ultramicroscopy 158, 1–7 (2015). 26093970 10.1016/j.ultramic.2015.06.010

[b44] Van Dyck D. & Chen F.-R. ‘Big Bang' tomography as a new route to atomic-resolution electron tomography. Nature 486, 243–246 (2012). 22699616 10.1038/nature11074

[b45] Meyer R. R., Kirkland A. I. & Saxton W. O. A new method for the determination of the wave aberration function for high resolution TEM. Ultramicroscopy 92, 89–109 (2002). 12138946 10.1016/s0304-3991(02)00071-2

[b46] Cosgriff E. C. . Image contrast in aberration-corrected scanning confocal electron microscopy. Adv. Imaging Electron Phys. 162, 45–76 (2010).

[b47] van Benthem K. . Three-dimensional ADF imaging of individual atoms by through-focal series scanning transmission electron microscopy. Ultramicroscopy 106, 1062–1068 (2006). 16875782 10.1016/j.ultramic.2006.04.020

[b48] Yang H. . Imaging screw dislocations at atomic resolution by aberration-corrected electron optical sectioning. Nat. Commun. 6, 7266 (2015). 26041257 10.1038/ncomms8266 PMC4468905

[b49] Ishikawa R., Lupini A. R., Hinuma Y. & Pennycook S. J. Large-angle illumination STEM: toward three-dimensional atom-by-atom imaging. Ultramicroscopy 151, 122–129 (2015). 25484363 10.1016/j.ultramic.2014.11.009

[b50] Ciston J. . Surface determination through atomically resolved secondary-electron imaging. Nat. Commun. 6, 7358 (2015). 26082275 10.1038/ncomms8358 PMC4557350

[b51] Kirkland E. J. Improved high resolution image processing of bright field electron micrographs. Ultramicroscopy 15, 151–172 (1984).

[b52] Strüder L. in Synchrotron Light Sources and Free-Electron Lasers 1–31Springer International Publishing (2015).

[b53] Ryll H. . A pnCCD-based, fast direct single electron imaging camera for TEM and STEM. J. Instrum. 11, P04006–P04006 (2016).

[b54] Schmidt J. . Extending the dynamic range of fully depleted pnCCDs. J. Instrum. 9, P10008–P10008 (2014).

