# Supplementary material for: Simultaneous atomic-resolution electron ptychography and Z-contrast imaging of light and heavy elements in complex nanostructures
Source: Nat Commun. 2016 Aug 26;7:12532. doi: 10.1038/ncomms12532 (PMC5007440; doi:10.1038/ncomms12532)
Supplement: Supplementary Information — Supplementary Figures 1-13, Supplementary Table 1, Supplementary Methods and Supplementary References. [file ncomms12532-s1.pdf]

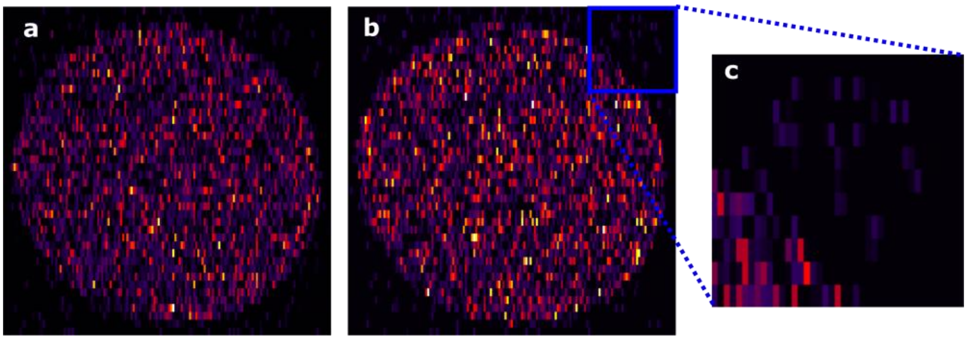

Supplementary Figure 1. Experimental convergent beam electron diffraction (CBED) patterns. **a, b** *Examples of individual frames of experimental CBED patterns recorded using a direct electron pixelated detector.* **c** *A magnified region of* **b** *showing single electron scattering events in the dark field. The detector is operated at a frame rate of 4000 frames per second, and the non-square pixels in the images are due to detector binning by a factor of 4 in the vertical direction to increase detector frame rate. The microscope was operated under a probe current of around 1.8 pA, and each frame of CBED pattern has approximately 2800 electrons.*

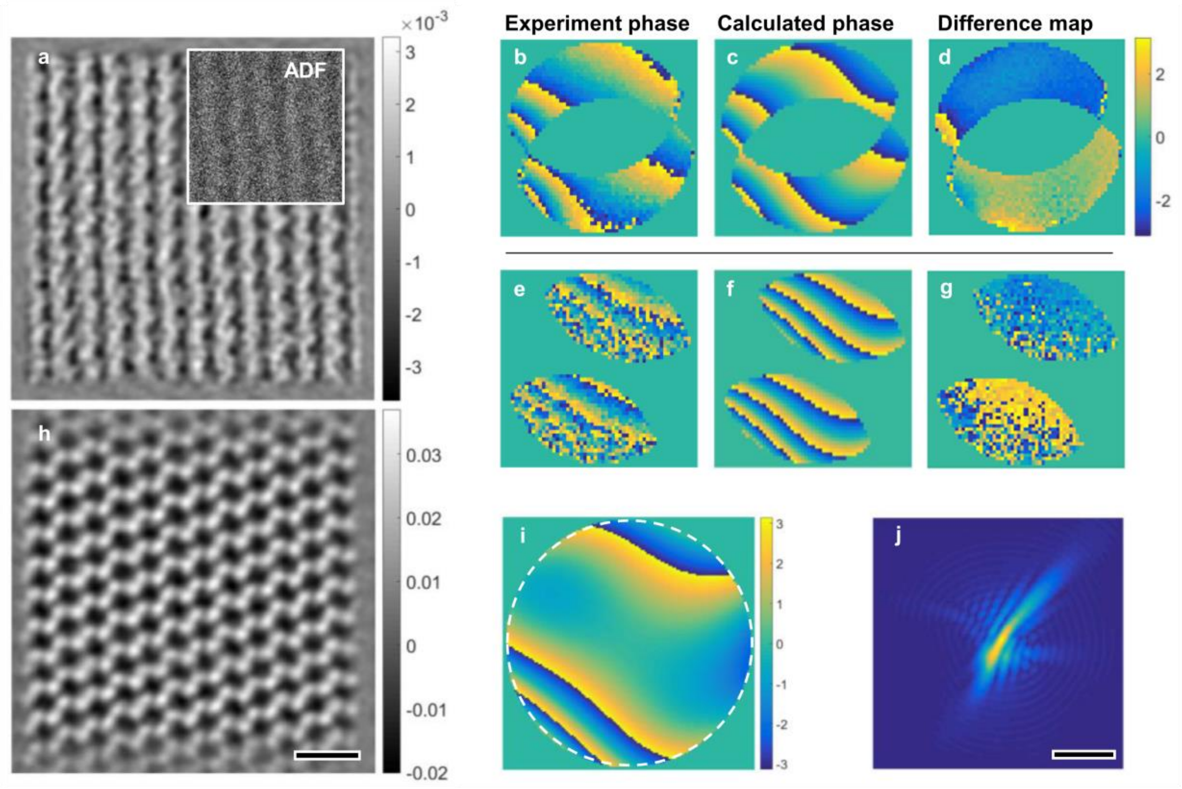

Supplementary Figure 2. Aberration free imaging through direct inversion of probe aberrations. **a** *Experimental result of the reconstructed phase and the simultaneous ADF image (inset) from single layer graphene in the presence of probe aberrations. The data presented here was collected in an aberration-corrected instrument with poorly aligned conditions. The disc double-overlap areas from* $G(\mathbf{K_f}, \mathbf{Q_p})$ *in the detector plane show non-flat phase surfaces due to lens aberrations, as shown in* **b** *and* **e** *for two example spatial frequencies* $\mathbf{Q_p}$*. The map of difference between the experimental phase and* **c**, **f** *the calculated phase arising from the measured lens aberrations, shows a rather flat phase surface in* **d** *and* **g** *as expected from an aberration free imaging condition.* **i** *The SVD diagnosed phase of the probe forming aperture is used as input in WDD to obtain* **h** *the phase of the object. The real-space intensity of the electron probe calculated from* **i**, *is shown in* **j**. *The scale bars in* **h** *and* **j** *are 0.5nm and 1nm, respectively and the colourbar units are in radians.*

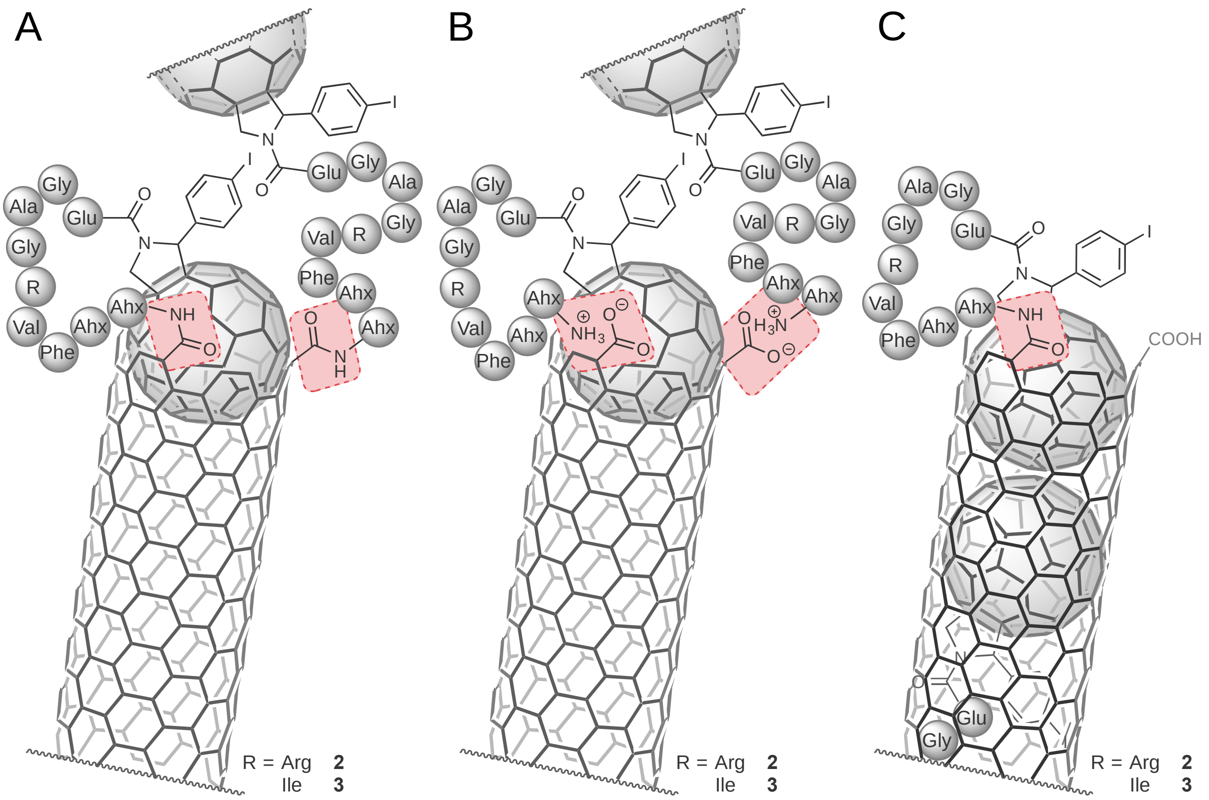

***Supplementary Figure 3**: **Potential carbon nanotube-peptide-fC_{60} product hybrid interactions. A**: Designed, intended structure of hybrids **2** and **3**. **B**: Possible ionic interactions. **C**: Aggregations and peapod formation.*

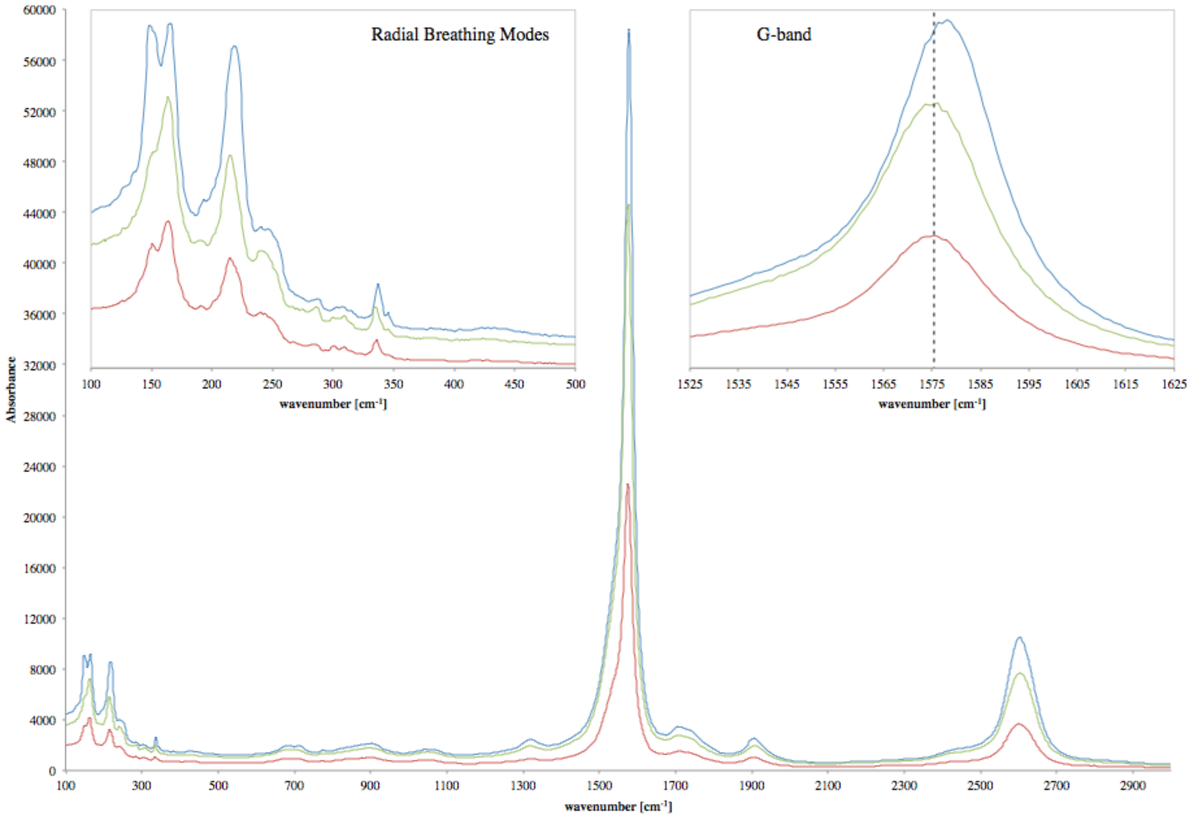

Supplementary Figure 4: Raman spectra of oxidised carbon nanotubes (1), 3 representative regions. *The peak ratio of the D-band (~1320 cm^{-1}), indicating defects in the tubular nanotube sidewall structure, and the G-band (~1575 cm^{-1}), reflecting the longitudinal stretch vibration of sp^{2}-hybridised carbons is small and confirms low sidewall destruction during the oxidation. The radial breathing mode resonances (left insertion) indicate the presence of single walled and double walled carbon nanotubes. The right insert shows the G-band with a red-shift of 8 cm^{-1} in one of the recorded spectra and reveals a dependency on the microenvironment of Raman spectroscopy.*

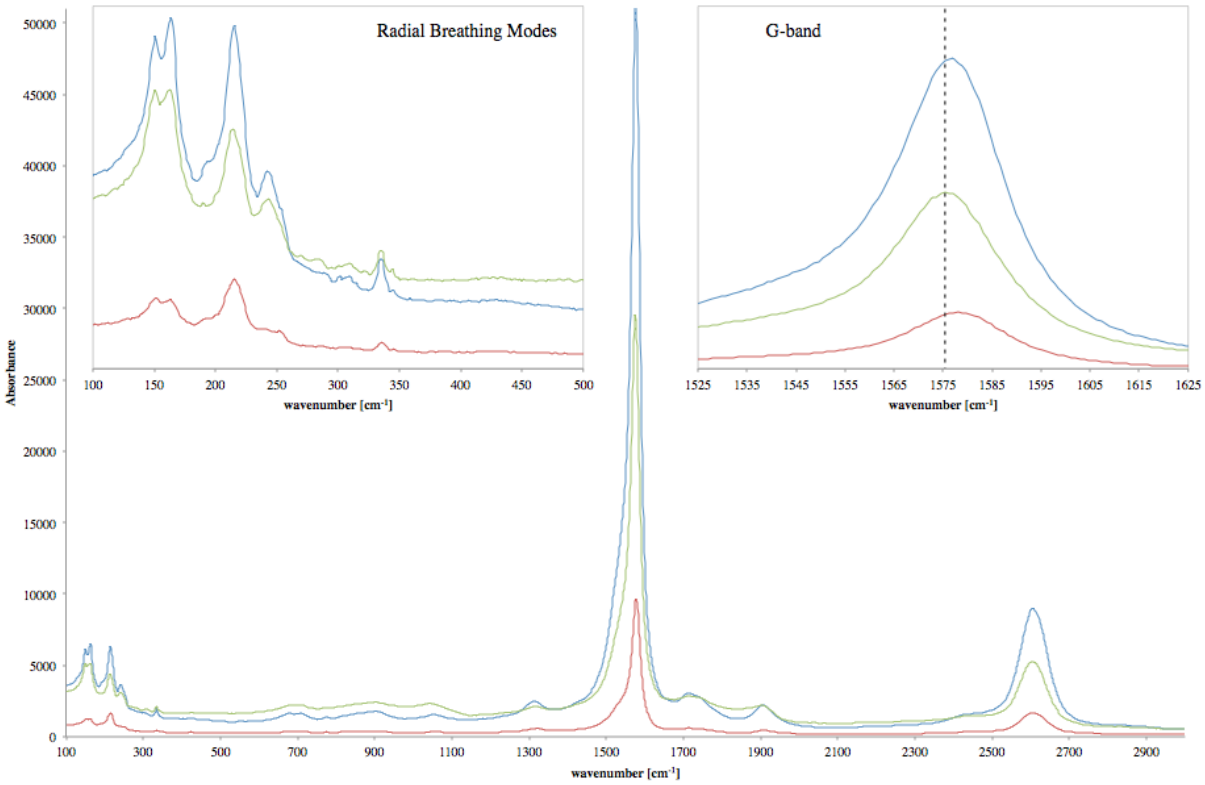

Supplementary Figure 5: Raman spectra of three representative surface regions of CNT-C_{60} construct 2*. The peak ratio of the D-band (~1320 cm^{-1}) to the G-band (~1575 cm^{-1}) is small, suggesting low sidewall destruction during the synthesis. The left insert shows the radial breathing modes; additional fullerene peaks or changes in the tubular structure can not be observed. The right insertion shows the G-band with a red-shift of up to 3 cm^{-1}, which is dependent on the recorded microenvironment and can not be ascribed to the presence of C_{60} as previously observed.*

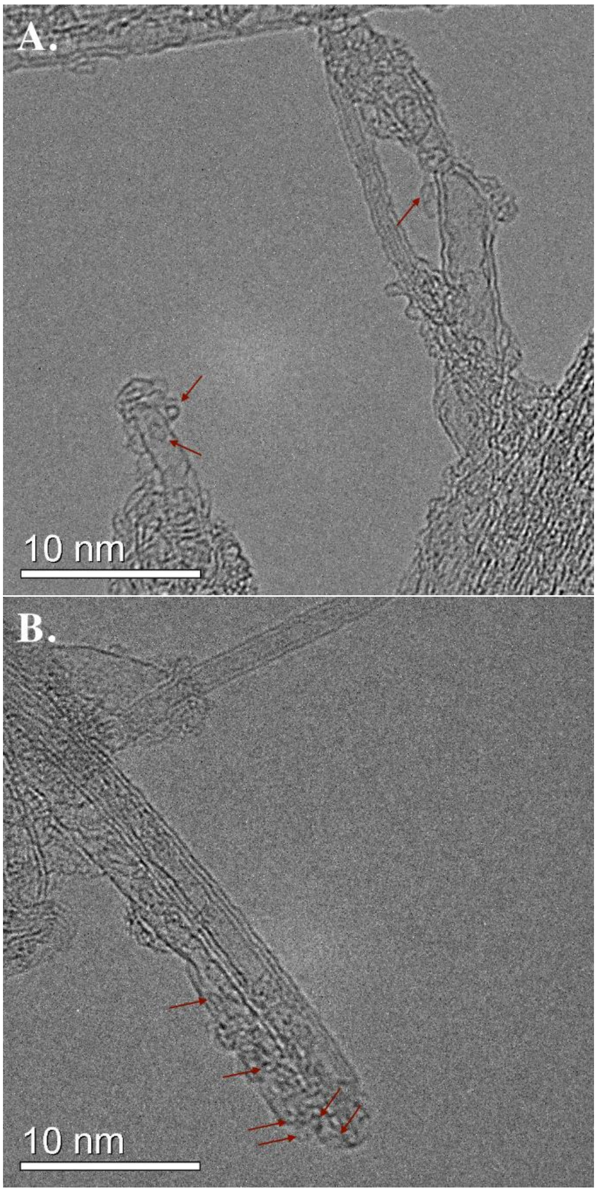

***Supplementary Figure 6**: **HRTEM of Hybrid 2 Sample**. **A**: A single walled carbon nanotube (SWNT) with at least two fullerenes at the open tube end. Due to focus limitations only two C_{60} were imaged (arrows indicating the circular shaped C_{60} of ø = 0,71 nm). One C_{60} is presumably inside the tube. In the image two double walled tubes (DWNT) which have been damaged by the electron beam can be seen. The arrow indicates a fullerene that is attached to a defect site in the tube structure. **B**: A SWNT, DWNT and triple walled NT can be observed. The arrows indicate presumed fC_{60} located at the SWNT end.*

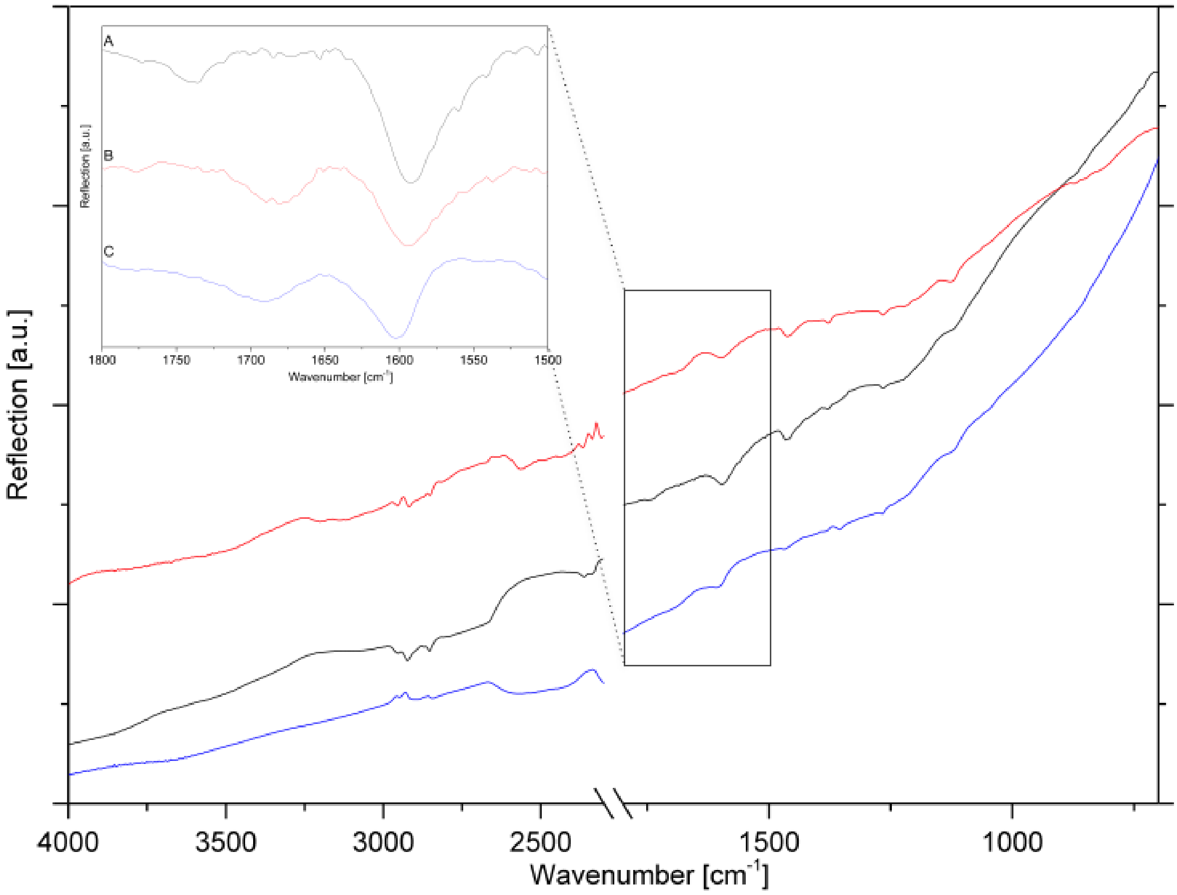

***Supplementary Figure 7**: **IR spectroscopy of 1 and Hybrids 2 and 3**. Bands of C=C (~1600 cm^{-1}) and CH (~2900 cm^{-1}) can be observed in CNT-COOH **1** (black trace), CNT-C_{60} construct **2** (red trace) and sample **3** (blue trace). Expansion suggests carbonyl C=O (~1740 cm^{-1}) in oxidised CNTs (insert, baseline corrected spectra, **A**) undergoes a blue-shift during functionalization to amide bonds (~1690 cm^{-1}) in the modified CNTs **2** (**B**) and **3** (**C**).*

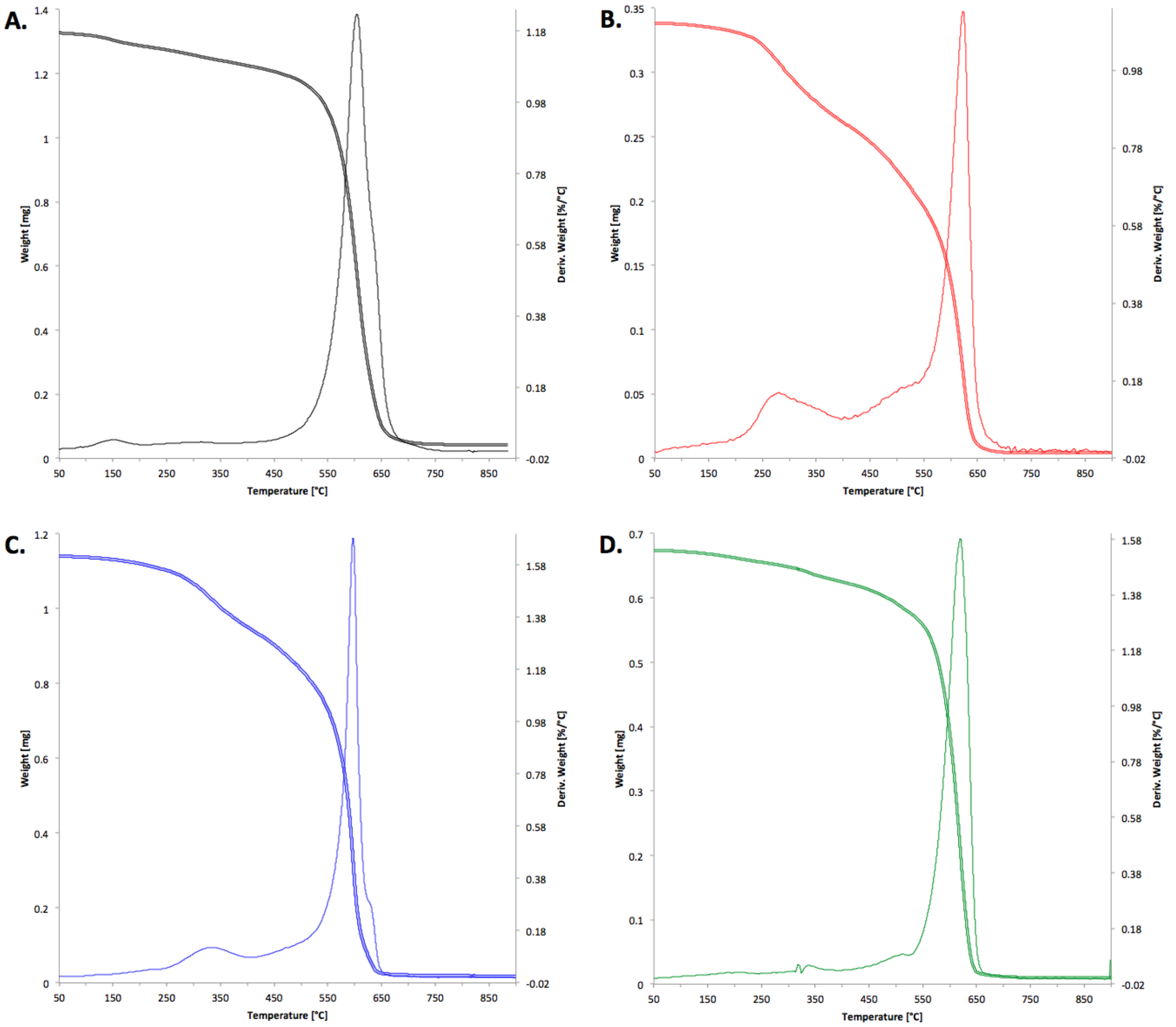

***Supplementary Figure 8: Thermogravimetric analysis traces and their first derivation. A**: Oxidized carbon nanotubes show combustion at 150 °C (water), 604 °C (CNT) and 634 °C. The amount of oxidized iron catalyst is marginal (3.15%); **B**: The TGA curve suggests four combustion temperatures for the CNT-C_{60} construct **2**; **C**: Sample of construct **3** suggests at least three oxidizable species; **D**: A mixture of physically mixed (but not reacted) peptide functionalized fullerene ($T_{combust.}$ = 336 and 512 °C) and nanotubes **1** (620 °C) as a 'spiked, dirty' comparison.*

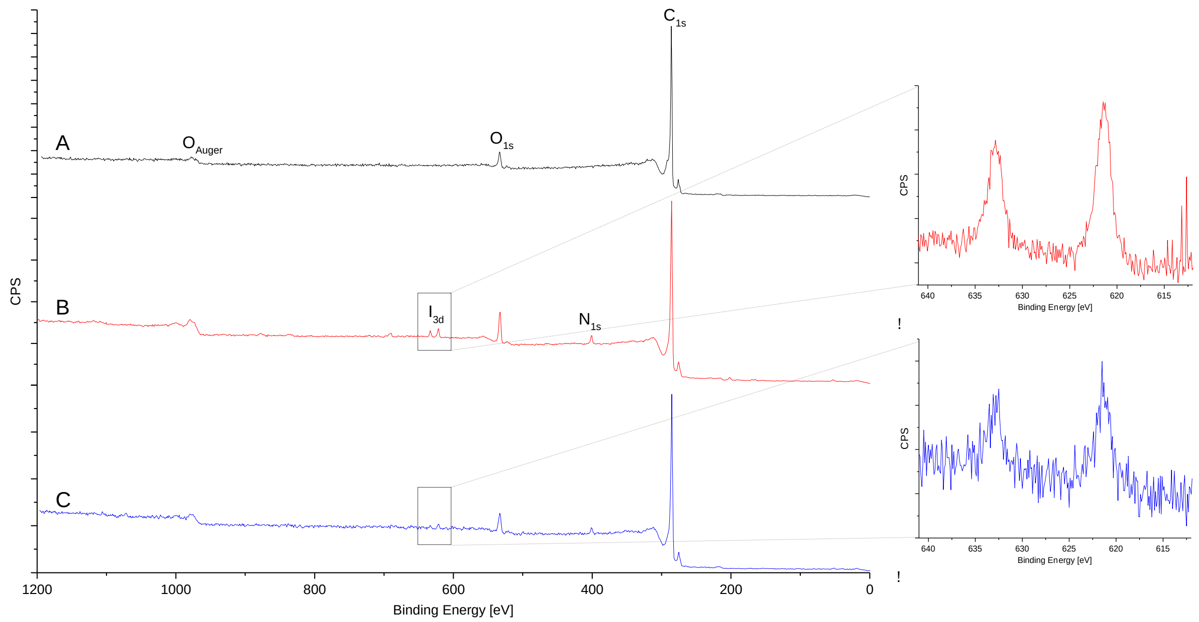

!**Supplementary Figure 9: Elemental composition of CNT samples by x-ray photoelectron spectroscopy.** *The XPS data showing the presence of carbon and oxygen for CNT-COOH* ***1*** *(**A**, black trace) and additional nitrogen and iodine due to the presence of peptide linked C_{60} in sample* ***2*** *(**B**, red trace) and* ***3*** *(**C**, blue trace). The I_{3d} peaks were used for semi-quantification of the modification yielding one iodine per 321 carbon atoms for* ***1****, which equals the functionalisation of every 13th carboxylic acid group, and one iodine every 613 carbon atoms for* ***2****, every 16th carboxylic acid group reacted.*

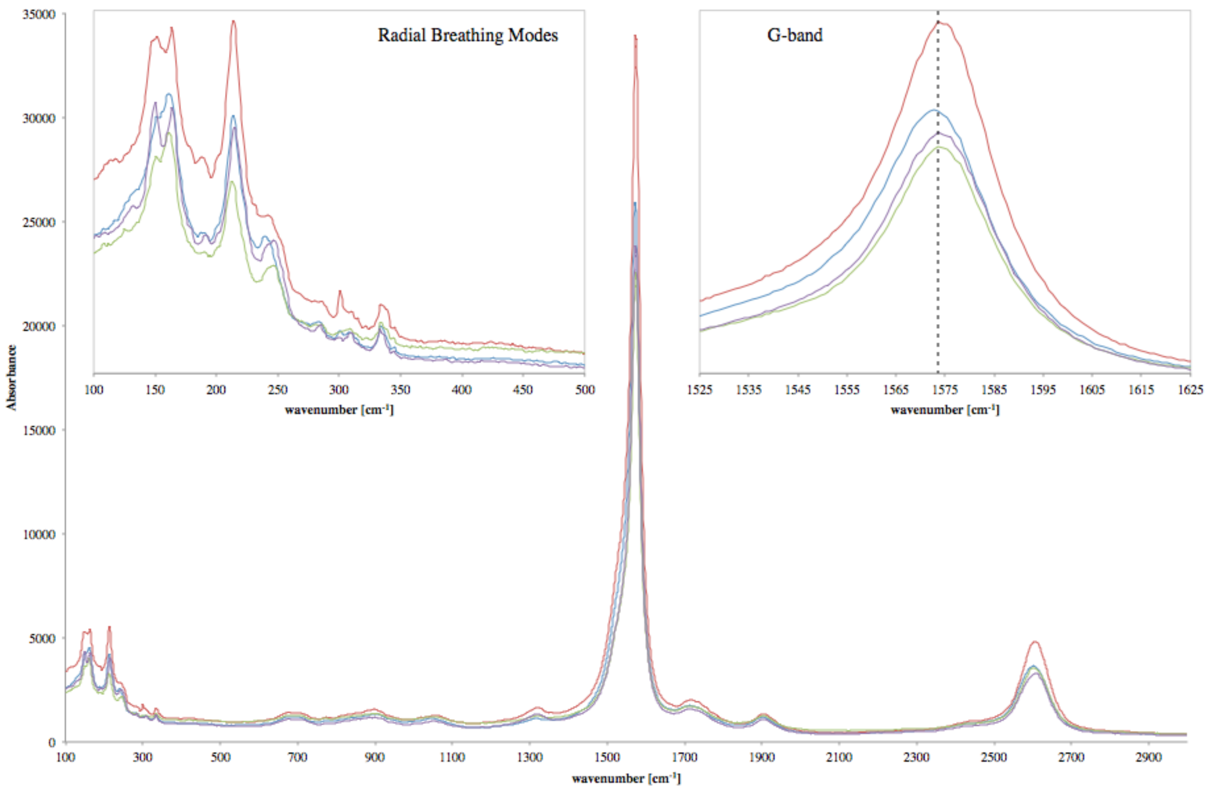

Supplementary Figure 10: *Four Raman spectra of CNT-C_{60} construct* ***3****. The peak ratio of the D-band (~1320 cm^{-1}) to the G-band (~1575 cm^{-1}) is low, suggesting small sidewall destruction during the synthesis. The left insert shows the radial breathing modes; fullerene peaks at 268 and 492 cm^{-1} are not observed. The right insertion shows the G-band without a red-shift, indicating no presence of fC_{60} as previously observed.*

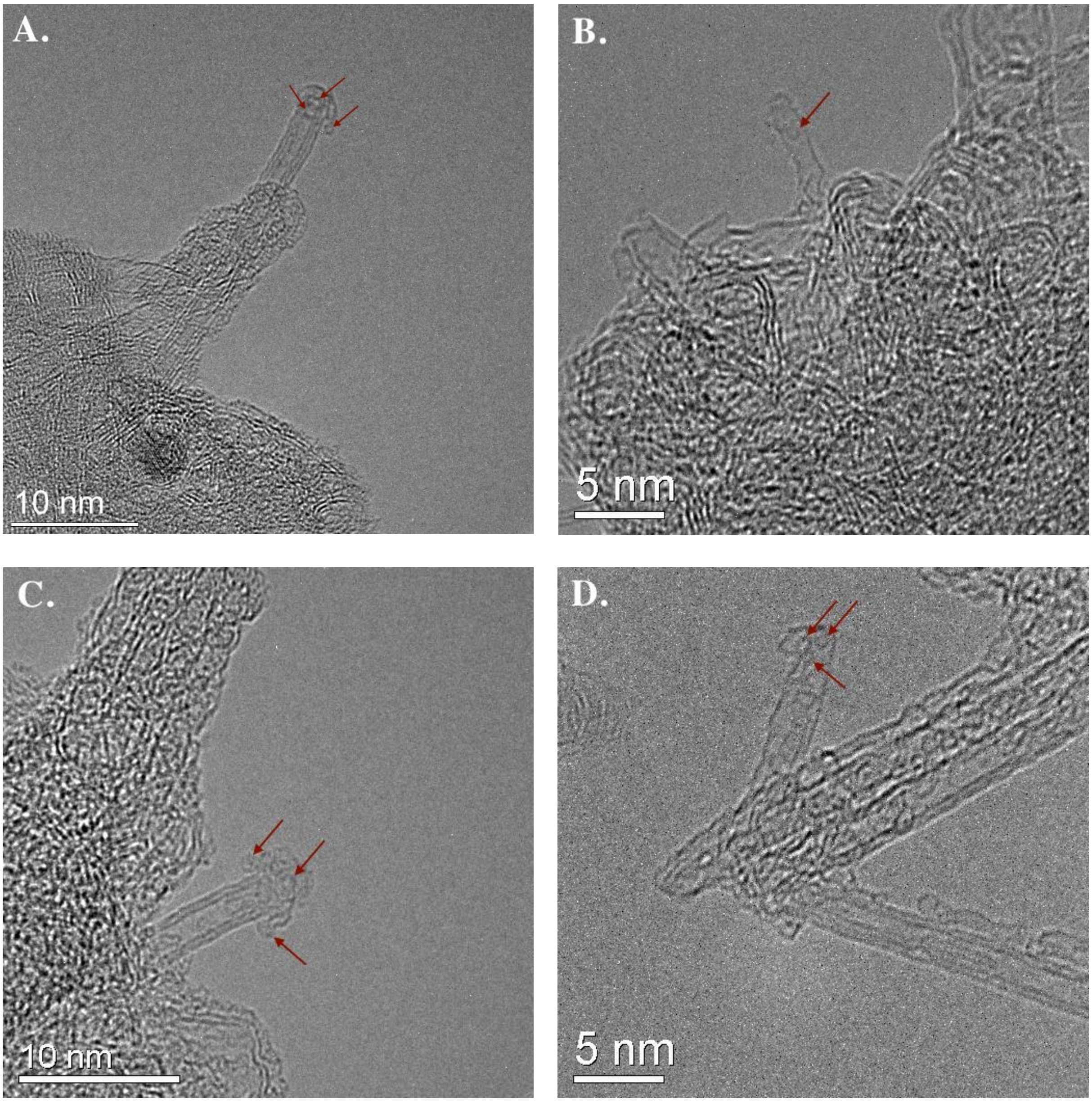

***Supplementary Figure 11: HRTEM of Hybrid 3 Samples**. Carbon nanotube end functionalisation with modified fullerenes (sample **3**) was visualized by means of HRTEM. Single, double and multi walled carbon nanotubes were observed. The presence of C_{60} molecules as indicated by the arrows, was discovered on all tube species preferentially associated with the tip regions, as designed. Carbon debris and focus adjustments of the flexible CNT ends complicated the interpretation of image **D**.*

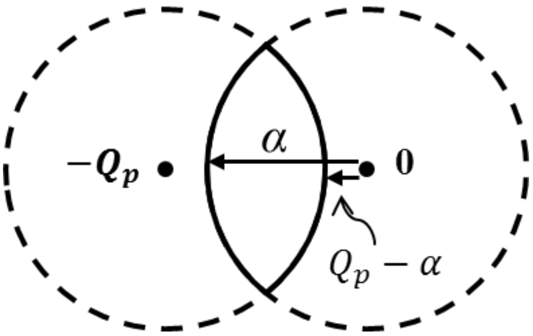

Supplementary Figure 12. The geometry of the disc overlap region in the detector plane ($\mathbf{K_f}$ space).
The maximum and minimum values of $\mathbf{K_f}$ in the direction parallel to $\mathbf{Q_p}$ are shown.

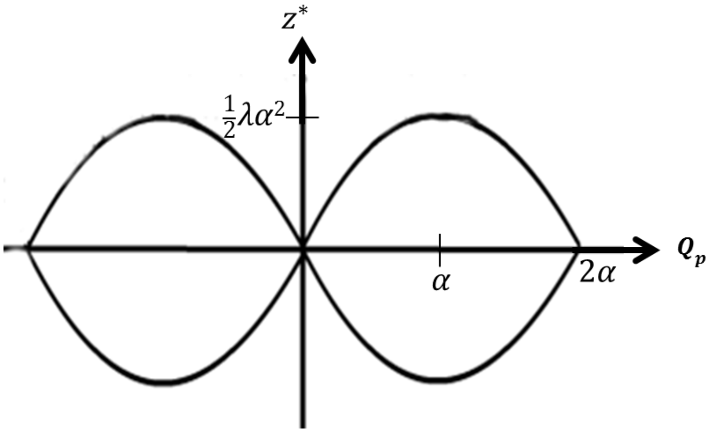

***Supplementary Figure 13. A cross-section through the bounds of the 3D contrast transfer** plotted as function of lateral spatial frequency $\mathbf{Q_p}$ and longitudinal spatial frequency z^*.*

Supplementary Table 1. *Measured values of the aberration coefficients of the graphene sample.*

Aberration Coefficients		Measured values
Defocus	C_1	-3.02 (nm)
Two-fold astigmatism	C_{12a}	+2.30 (nm)
Two-fold astigmatism	C_{12b}	+6.61 (nm)
Three-fold astigmatism	C_{23a}	-13.7 (nm)
Three-fold astigmatism	C_{23b}	-70.4 (nm)
Axial coma	C_{21a}	182 (nm)
Axial coma	C_{21b}	-128 (nm)
Spherical aberration	C_3	-4.94 (µm)
Four-fold astigmatism	C_{34a}	+2.28 (µm)
Four-fold astigmatism	C_{34b}	+0.0620 (µm)
Star aberration	C_{32a}	+2.87 (µm)
Star aberration	C_{32b}	-1.25 (µm)

Supplementary Methods

1. Measuring Lens Aberrations through SVD Matrix Inversion.

Because WDD allows residual lens aberrations to be corrected from the 4D data-set, the ability of measuring residual lens aberrations within the 4D data-set becomes particularly beneficial and leads towards aberration free phase imaging. In this section, we introduce an aberration measurement algorithm that measures the aberration coefficients of low and high orders by solving a set of linear equations through a deterministic matrix inversion using singular value decomposition (SVD)[1]. This method makes use of the phase information inside the disc-overlap region, and can be applied to both crystalline and noncrystalline specimens.

Before going through the details of the aberration measurement algorithm, Supplementary Fig.2 demonstrates an example of measuring and correcting lens aberrations using a sample of single layer graphene with an electron probe arising from a poorly aligned aberration corrector. The poor quality of the ADF image and the initial phase reconstruction assuming no aberrations (Supplementary Fig.2a) means that data like this would usually be discarded. By applying the SVD, aberration coefficients up to 3rd order were measured (see Supplementary Table 1). The quality of the aberration measurements can be seen from two examples of the overlapping discs showing the good match between the experimental phases and the calculated phases arising from only the measured aberration coefficients (compare Supplementary Fig.2b with 2c, and 2e with 2f). In the Supplementary Fig.2d,g, the maps of difference also show a reasonably flat phase surface, indicating an efficient aberration compensation has been achieved. The phase in the probe-forming aperture in Supplementary Fig.2i and the real space probe in Supplementary Fig.2j calculated using the measured aberration coefficients were directly fed into the WDD to obtain Supplementary Fig.2h an aberration free phase image with significantly improved resolution and quantitative phase values compared to the initial reconstruction in Supplementary Fig.2a.

1.1 Mathematical description of the phase in the disc-overlap region

If we assume the weak-phase object approximation then,

$$\Psi(\mathbf{K}) = \delta(\mathbf{K}) + \Psi'(\mathbf{K}) \tag{1}$$

where $\Psi'(\mathbf{K}) \ll 1$ and δ is the Dirac delta function. Substituting equation 1 into equation 2 of the main text gives

$$G(\mathbf{K_f}, \mathbf{Q_p}) = |A(\mathbf{K_f})|^2 \delta(\mathbf{Q_p}) + A(\mathbf{K_f}) A^*(\mathbf{K_f} + \mathbf{Q_p}) \Psi'^*(-\mathbf{Q_p}) + A^*(\mathbf{K_f}) A(\mathbf{K_f} - \mathbf{Q_p}) \Psi'(\mathbf{Q_p}). \tag{2}$$

equation 2 represents, for $\mathbf{Q_p} \neq \mathbf{0}$, the appearance of two disc overlap regions as shown in Fig. 1d,e of the main text. The phase $\angle G(\mathbf{K_f}, \mathbf{Q_p})$ in the two double disc-overlap regions in the detector plane can be described as

$$\angle G(\mathbf{K_f}, \mathbf{Q_p}) = [\chi(\mathbf{K_f}) - \chi(\mathbf{K_f} + \mathbf{Q_p}) - \angle\Psi'(-\mathbf{Q_p})] + [\chi(\mathbf{K_f} - \mathbf{Q_p}) - \chi(\mathbf{K_f}) + \angle\Psi'(\mathbf{Q_p})] \tag{3}$$

where $\chi(\mathbf{K_f})$ is the aberration function and $\angle$ is the phase angle operator. The two terms in the brackets describe the two disc-overlap regions formed by -$\mathbf{Q_p}$ and +$\mathbf{Q_p}$, respectively.

Following the Krivanek notation[2], the aberration function up to third order in Cartesian coordinates is given by:

$$\chi(\mu,\nu) = \frac{2\pi}{\lambda}[+\frac{1}{2}(C_1(\mu^2+\nu^2)+C_{12a}(\mu^2-\nu^2)+2C_{12b}\mu\nu)$$
$$+\frac{1}{3}\left(C_{23a}(\mu^3-3\mu\nu^2)+C_{23b}(3\mu^2\nu-\nu^3)+C_{21a}(\mu^3+\mu\nu^2)+C_{21b}(\nu^3+\mu^2\nu)\right)$$
$$+\frac{1}{4}\left(C_3(\mu^4+\nu^4+2\mu^2\nu^2)+C_{34a}(\mu^4-6\mu^2\nu^2+\nu^4)+C_{34b}(4\mu^3\nu-4\mu\nu^3)+C_{32a}(\mu^4-\nu^4)+\right.$$
$$\left.C_{32b}(2\mu^3\nu+2\mu\nu^3)\right)] \qquad (4)$$

where $[\mu,\nu]$ is the scattering vector in Cartesian coordinates. Here the first order of μ,ν terms describing the probe positions are not included. The aberration function equation 4 can be rewritten as follows for simplicity,

$$\chi(\mu,\nu) = \widetilde{a_1}(\mu,\nu)C_1 + \widetilde{a_2}(\mu,\nu)C_{12a} + \widetilde{a_3}(\mu,\nu)C_{12b} \ldots + \widetilde{a_{12}}(\mu,\nu)C_{32b}$$

where $\widetilde{a_1}(\mu,\nu)$ to $\widetilde{a_{12}}(\mu,\nu)$ are functions of the scattering vector $[\mu,\nu]$ as described in equation 3.

Taking one side ($-\mathbf{Q_p}$) of the two double-overlap regions, the phase can be analytically expressed as a linear equation of all the aberration coefficients and the phase of the specimen as following,

$$\chi(\mathbf{K_f})-\chi(\mathbf{K_f}+\mathbf{Q_p})-\angle\Psi(-\mathbf{Q_p}) = \left(\widetilde{a_1}(K_\mu,K_\nu)-\widetilde{a_1}(K_\mu+Q_\mu,K_\nu+Q_\nu)\right)C_1+\cdots+$$
$$\left(\widetilde{a_{12}}(K_\mu,K_\nu)-\widetilde{a_{12}}(K_\mu+Q_\mu,K_\nu+Q_\nu)\right)C_{32b}-\angle\Psi(-\mathbf{Q_p}) \qquad (5)$$

where $\mathbf{K_f}=[K_\mu,K_\nu]$ and $\mathbf{Q_p}=[Q_\mu,Q_\nu]$ are vectors expressed in Cartesian coordinates along axes parallel to the Cartesian vectors $\hat{\mu}$ and $\hat{\nu}$.

Note that the $+\mathbf{Q_p}$ side of the two double-overlap regions can be treated exactly the same way throughout the whole process starting from equation 3, but will not be described in this text.

1.2 Matrix representation of the linear equations

In a ptychographic 4D dataset, for example an experiment dataset with 256x256 probe positions and 264x264 detector pixels, there are a large number of $(\mathbf{K_f},\mathbf{Q_p})$ observations that are located within the double-overlap regions (orders of magnitude larger than the number of unknown aberration coefficients), leading to a large set of linear equations as in equation 5. This set of linear equations can be written in a matrix format with each term being defined as following,

$$\mathbf{b} = \tilde{A}(\mathbf{K_f},\mathbf{Q_p})\cdot\boldsymbol{x} \qquad (6)$$

$$\tilde{A}(\mathbf{K_f},\mathbf{Q_p}) =$$
$$\begin{pmatrix} \widetilde{a_1}(K_\mu,K_\nu)-\widetilde{a_1}(K_\mu+Q_{\mu1},K_\nu+Q_{\nu1}), & \cdots & \widetilde{a_{12}}(K_\mu,K_\nu)-\widetilde{a_{12}}(K_\mu+Q_{\mu1},K_\nu+Q_{\nu1}), 1\ldots & 0\ldots & 0 \\ \vdots & \ddots & \vdots & \vdots \quad \ddots & \vdots \\ \widetilde{a_1}(K_\mu,K_\nu)-\widetilde{a_1}(K_\mu+Q_{\mu k},K_\nu+Q_{\nu k}), & \cdots & \widetilde{a_{12}}(K_\mu,K_\nu)-\widetilde{a_{12}}(K_\mu+Q_{\mu k},K_\nu+Q_{\nu k}), 0\ldots & 0\ldots & 1 \end{pmatrix}_{M\times N}$$
(7)

$$\boldsymbol{x} = \left[C_1, C_{12a}, C_{12b}, C_{23a}, \ldots\ldots, C_{32a}, C_{32b}, -\angle\Psi(-\mathbf{Q_{p1}}), \ldots -\angle\Psi(-\mathbf{Q_{pk}})\right]_{N\times1} \qquad (8)$$

$$\mathbf{b} = \left[\angle G(\mathbf{K_f},\mathbf{Q_{p1}}), \ldots \angle G(\mathbf{K_f},\mathbf{Q_{p2}}), \ldots \angle G(\mathbf{K_f},\mathbf{Q_{pk}})\right]_{M\times1} \qquad (9)$$

Again, the above matrix representation describes only one side ($-\mathbf{Q_p}$) of the two double-overlap regions, but the other side ($+\mathbf{Q_p}$) can be treated in exactly the same way, and the two matrices from the two double-overlap regions can be combined for direct matrix inversion.

1.3 Singular value decomposition

Solving the unknown aberration coefficients can therefore be performed by solving the set of linear equations as follows through direct matrix inversion. As discussed above, because the number of observations *M* in a hugely redundant 4D dataset is much larger than the number of unknown parameters *N*, the matrix $\tilde{A}$ is over-determined. Inversion of matrix $\tilde{A}$ can be solved using a method called singular value decomposition. A detailed description of SVD will not be shown in this text, and the reader is referred to the literature for example in [1] for more details.

1.4 Matrix preparation and Selection of $\mathbf{Q_p}$

Even though the $\angle G(\mathbf{K_f}, \mathbf{Q_p})$ in the double-overlap regions from the entire range of $\mathbf{Q_p} < 2\alpha$ can be used in the linear equations, practically it's sufficient to use only a number of $\mathbf{Q_p}$ values whose $\angle G(\mathbf{K_f}, \mathbf{Q_p})$ have the best signal-to-noise ratio, which corresponding to those with the largest modulus of $G(\mathbf{K_f}, \mathbf{Q_p})$ summed over $\mathbf{K_f}$. Therefore, the selection of $\mathbf{Q_p}$ can be fully automated by ranking the modulus of $G(\mathbf{K_f}, \mathbf{Q_p})$ summed over $\mathbf{K_f}$. An important requirement for the selection of $\mathbf{Q_p}$ is to make sure $\mathbf{Q_p}$ vectors of different directions are selected so that astigmatism aberrations can be effectively measured.

1.5 Iterative algorithm for solving aberrations from low to high orders

Solving the set of linear equations involves a step to unwrap the phase surface of $\angle G(\mathbf{K_f}, \mathbf{Q_p})$ if the values go beyond 2π. Ideally if the unwrapping is perfect, all of the $\mathbf{Q_p}$ values can be used for matrix inversion, and all of the low and high aberration coefficients can be solved instantly using SVD without the use of an iterative approach. However, when working with low dose datasets with a low signal-to-noise ratio, unwrapping the phase becomes non-trivial. This problem can be solved by adopting an iterative approach to measure the low order aberrations and compensate the phase due to low order aberrations first, which iteratively helps to "flatten" the phase surface and reduce the errors induced by phase unwrapping, thus leading to a more precise measurement of higher order aberrations iteratively. The iteration procedure can be described as following:

Step 1: Prepare matrix $\tilde{A}$ using equation 6, from *k* number of spatial frequencies from $\mathbf{Q_{p1}}$ to $\mathbf{Q_{pk}}$ and the scattering vector $\mathbf{K_f}$ values located inside the disc overlap regions under each spatial frequency.

Step 2: Start with iteration number $j = 1$. Initialize $\boldsymbol{x}_{j-1} = \boldsymbol{x}_{\mathbf{0}}$ following equation 7 and set all the aberration coefficients inside $\boldsymbol{x}_0$ to zero. Initialize $\mathbf{b}_{j-1} = \mathbf{b}_0$ as described in equation 8.

Step 3: $\Delta\boldsymbol{x}_j$ is defined as $\tilde{A} \cdot \Delta\boldsymbol{x}_j = \mathbf{b}_{j-1}$. Calculate $\Delta\boldsymbol{x}_j$ through the SVD matrix inversion[1]. Retain only 1st order aberrations values in $\Delta\boldsymbol{x}_j$, and setting the higher orders to zero.

Step 4: Update $\boldsymbol{x}_j = \boldsymbol{x}_{j-1} + \beta \cdot \Delta\boldsymbol{x}_j$, where β is the updating step parameter whose maximum value is 100%. In this experiment, we set $\beta = 0.5$ to apply 50% correction in each iteration.

Step 5: Calculate the overlapping discs $A(\mathbf{K_f})A^*(\mathbf{K_f} + \mathbf{Q_p})_{\boldsymbol{x}_j}$ using the aberrations $\boldsymbol{x}_j$.

Step 6: Calculate the compensated phase $\angle G(\mathbf{K_f}, \mathbf{Q_p})_{\mathrm{cmp}}$ using the experimental phase $\angle G(\mathbf{K_f}, \mathbf{Q_p})_{\mathrm{ex}}$ and the aberration coefficients $\boldsymbol{x}_j$ as following and unwrap the phase.

$$\angle G(\mathbf{K_f}, \mathbf{Q_p})_{\mathrm{cmp}} = \angle\left[\left|G(\mathbf{K_f}, \mathbf{Q_p})_{\mathrm{ex}}\right| \cdot e^{i \cdot \angle G(\mathbf{K_f}, \mathbf{Q_p})_{\mathrm{ex}}} \cdot e^{-i \cdot \angle\left(A(\mathbf{K_f}) A^*(\mathbf{K_f}+\mathbf{Q_p})_{x_j}\right)}\right] \tag{10}$$

Step 7: Set the iteration number $j = j + 1$. Prepare $\boldsymbol{b}_{j-1} = [\angle G(\mathbf{K_f}, \mathbf{Q_p})_{cmp}, \ldots]$ the same way as in equation 8 but use the compensated phase values instead of the experimental phase values.

Step 8: Iterate Step 3 to Step 7, and gradually apply correction to include higher order aberrations in step 3 if $\Delta \boldsymbol{x}_j$ is smaller than threshold and a convergence is reached.

2. Specimen preparation and Characterization.

2.1 Synthesis and Characterization Methods

^{1}H NMR spectra were recorded at room temperature on the following spectrometers: 200 MHz: Bruker DPX200, 400 MHz: Bruker DQX400 and AV400, 500 MHz: Bruker AV500. Unless reported otherwise, spectra were recorded in $CDCl_3$ solution. Chemical shifts are reported in δ units relative to $CHCl_3$ (δ_H = 7.26). The following abbreviations were used throughout: s =singlet, d = doublet, dd = doublet of doublets, dt = doublet of triplets, t = triplet, tt = triplet of triplets, q = quartet, quin = quintet, m = multiplet.

^{13}C NMR spectra were recorded at room temperature on the following spectrometers: 100 MHz: Bruker DQX400, 125 MHz: Bruker DRX500. Unless reported otherwise, spectra were recorded in $CDCl_3$ solution. Chemical shifts are reported in δ units relative to $CDCl_3$ (δ_C = 77.23 central line of triplet).

^{19}F NMR spectra were recorded at room temperature a 376 MHz on a Bruker AVIII HD 400. Unless reported otherwise, spectra were recorded in $CDCl_3$ solution. Chemical shifts are reported in δ units

HRTEM: SWNT samples and hybrids were dispersed in EtOH [10 µg/mL], sonicated for 30 to 60 min, placed dropwise (4 x 6 µL) onto a Lacey carbon copper support grid and dried by evaporation. The JEOL JEM-3000F with a field-emission gun operated at 300 kV. The microscope was equipped with a 1k Gatan 794 MultiScan camera. The data were analyzed with DigitalMicrograph.

EDX: An Oxford Instruments EDS detector equipped with an ultra-thin polymer window and controlled by Oxford Instruments Isis software INCA was used for the acquisition on the JEOL JEM-3000F.

XPS: Data were collected under high vacuum (approx. 10^{9} torr) on a VG Escalab XPS Spectrometer by Ashley Shepherd in the Surface Analysis Facility, Chemistry Research Laboratory. Analysis was undertaken with CasaXPS software.

TGA: SWNT samples were measured in synthetic air flow (20 mL/min) using the model STA 449 F1 Jupiter® from Netzsch by Magdalena Kierkowicz, Institut de Ciència de Materials de Barcelona (ICMAB). An initially isothermal run profile was maintained for 15 min and then increased at 10 °C/min to 900 °C.

HRMS: Data was determined under conditions of ES^{+} on a Micromass Q-Tof micro (resolution = 4 x 10^{3} D) using H_3PO_4-clusters as a lock-mass in positive ion mode.

HPLC: was performed on a Dionex Ultimate 3000 system. Gradients were established for a Phenomenex Jupiter 4u Proteo 90A (250 x 4.6 mm) and Synergi 4u Hydro-RP 80A (100 x 4.6 mm, 4 micron and 100 x 21.2 mm, 4 micron). The solvent system was mixed appropriately from H_2O (MilliQ purity) and MeCN, 0.1% formic acid (FA) each.

MALDI-ToF spectra were recorded on a Waters® Micromass® MALDI micro MX™ Mass Spectrometer in the positive reflectron mode. The data were analysed with MassLynx V4.1.

Infrared spectra for small organic molecules were recorded on a Bruker Vector 22 IR spectrometer with the sample being pressed into KBr pellets. The IR of CNT samples were recorded using attenuated total reflection Fourier-transformed infrared (ATR-FTIR) on a Varian FTS-7000 Spectrometer and a DuraSamplIR II diamond crystal by Dr. Robert Jacobs in the Surface Analysis Facility, Chemistry Research Laboratory.

Raman spectra were recorded on a Jobin Yvon spectrometer (Horiba) equipped with a microscope, through a 50-fold magnification objective (Olympus Co.), by combining three sets of spectra. A 20 mW He-Ne laser (632 nm) was used. The 1800 L/mm grating provides a resolution starting from 1.0 cm^{-1} at 100 cm^{-1} up to 0.5 cm^{-1} at 3000 cm^{-1}. The abscissa was calibrated with a silicon standard. Raman spectra were directly taken from solid samples.

Reagents and Solvents: All reagents and solvents were obtained from Sigma-Aldrich, Alfa Aesar, Carbosynth, GLS Biochemicals, Fluka, and Frontier Scientific and were used directly as supplied, unless otherwise reported. Anhydrous solvents were bought from Sigma-Aldrich. All non-aqueous reactions were performed in oven- or flame- dried apparatus under argon or nitrogen atmosphere

using anhydrous solvents. Carbon nanotubes were supplied by Thomas Swan (produced using iron as a catalyst *via* arc-discharge method[3,4]) and steam-purified by Dr. Gerard Tobias, ICMAB[5,6].
Reaction Techniques: Reactions were monitored by thin layer chromatography on pre-coated aluminium-backed plates (Merck Kieselgel 60 with fluorescent indicator UV_{254}). Spots were visualised by quenching of UV fluorescence and/or by staining with potassium permanganate, iodine, ninhydrin, *p*-anisaldehyde or vanillin. Flash column chromatography was performed according to the method described by Still, Kahn and Mitra[7] with silica gel 60 (0.040-0.063 mm) (Geduran® Si 60) applying head pressure by means of manual flushing.

2.2 Hybrid Synthesis Overview

The synthesis of a hybrid, tethered system as envisaged in Fig.2 of the main manuscript was attempted as detailed below. Carbon nanotubes (CNT) were oxidized to introduce carboxylic acid groups for functionalization following literature procedures[8–11]. For clarity, samples are referred to number **1**, **2** and **3** through the main text and supplementary information, and their structures are shown in Fig.2 of the main manuscript, Supplementary Scheme S1 and Supplementary Scheme S2**.** The analysis of the resulting complex, product carbon nanomaterial was undertaken by means of infrared (IR, Supplementary Fig. 7) and Raman spectroscopy (see Supplementary Fig. 4, 5 and 10, respectively), high resolution transmission electron microscopy (HRTEM as shown in Supplementary Fig. 6 and 11), energy dispersive x-ray spectroscopy (EDX), X-ray fluorescence spectroscopy (XRF), X-ray photoelectron spectroscopy (XPS, Supplementary Fig. 9) and thermogravimetric analysis (TGA, Supplementary Fig. 8).
Characterizations by these conventional techniques suggested **fC_{60}**-functionalized carbon nanotube hybrids, as intended. These constructs were subjected to scanning transmission electron microscopy (STEM) with implemented ptychography to resolve the structure making use of the 'iodine-tagged' fullerene. In both representative samples **2** and **3**, **'**peapod' structures were observed, indicating the presence of incomplete covalent functionalization. We speculatively conclude that the CNT-peptide-C_{60} product mixtures comprise a mixture of covalently-attached fullerenes (Supplementary Fig.3A), potential ionic interactions[12–14] of CNT carboxylic acids with amine and guanidine groups in the peptide linker (Supplementary Fig.3B), and van der Waals-forced 'peapod'-type interactions (Supplementary Fig.3C).

2.3 Synthesis of CNT-COOH (1)

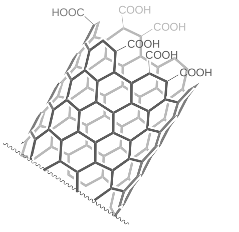

The suspension of CNTs (56.1 mg) in HNO_3 (8 M, 25 mL) was sonicated for 1.5 h. The mixture was filtered (0.2 µm polycarbonate membrane) and washed with H_2O_{dest} (2 x 250 mL). The CNTs were taken up in $NaOH_{aq}$ (4 M, 50 mL), sonicated for 15 min, filtered and washed with H_2O_{dest}. The NaOH-wash was repeated twice with sonication for 30 min and washing with H_2O_{dest}. The nanotubes were taken up in H_2O_{dest} (70 mL), sonicated for 30 min and filtered and washed with H_2O_{dest} until pH 7. The solid was taken up in $HCl_{aq.}$ (1 M, 50 mL), sonicated for 30 min, filtered and washed with H_2O_{dest} (250 mL), taken up in H_2O_{dest} (80 mL), sonicated for 30 min and filtered and washed with H_2O_{dest} until pH 5. The black solid was dried *in vacuo* at 60 °C to give oxidised CNTs CNT-COOH (**1**) (47.7 mg). **ATR-FTIR** ν_{max} 2953, 2922, 2868 (CH, CH_2), 2411 (OH, NH), 1755 (C=O acid), 1653 (sh), 1593 (C=C), 1454, 1178, 1079, 1028, 706; **Raman** A_D/A_G = 0.1232 (+2%); $\tilde{\nu}_{max}$ [cm^{-1}] 148, 161, 192, 214, 241, 285, 337, 345, 685, 898, 1055, 1320, 1576, 1709, 1906, 2606; **HRTEM** fairly clean, some carbon debris; **XRF** Cl, Ar, Ca, Cr, Fe, Ni detected; **XPS** O and C present; 1 x O per 18 carbon; **TGA** (in air) $T_{combust.}$ = 150.8, 603.8, 634(sh) °C, 3.14% residue; **TGA** (in N_2) $T_{combust.}$ = 355.4, 748.5 °C, 3.6% weight loss at 500 °C. The detailed synthesis methods refer to the literature [8–11].

2.4 Attempted Synthesis of Hybrid 2: XXFVRGAGQ peptide linking C_{60} to CNT-COOH

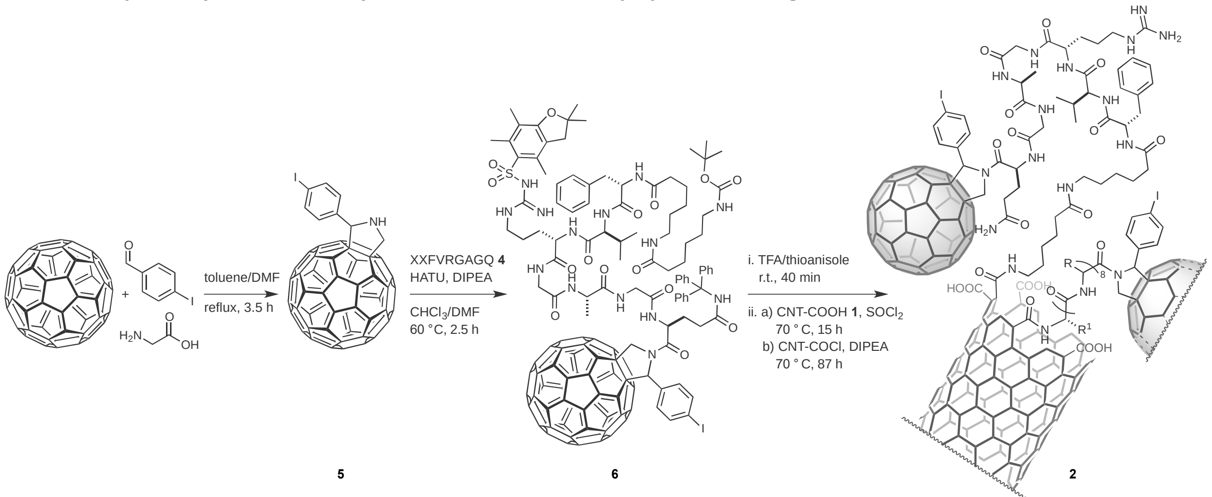

5 6 2

Supplementary Scheme S1**: Synthesis route of CNT-peptide-C_{60} construct **2**. Functionalisation of C_{60} gives the fulleropyrrolidine **5** and coupling thereof to peptide **4** gives cork **6.

2.4.1 NHBoc-Ahx-Ahx-Phe-Val-Arg(pbf)-Gly-Ala-Gly-Gln(trt)-OH (4)[15]

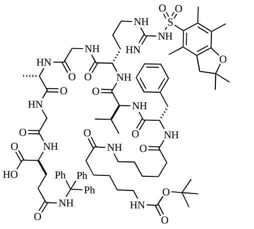

Preloading To the HMBA-AM resin (1.05 g) in DMF (2 mL) was added a filtered solution of Fmoc-Gln(trt)-OH (1.78 g, 2.92 mmol, 3 eq.), *N*,*N'*-dicyclohexyl carbodiimide (803.8 mg, 3.90 mmol, 4 eq.) and 4-(dimethyl-amino) pyridine (11.9 mg, 90 µmol, 0.1 eq.) in DMF (5 mL). After shaking for 2 h, the resin was washed with DMF (2 x 12 mL) and the coupling repeated. The resin was washed with DMF (5 x 12 mL). To the resin in DMF (7 mL) was added acetic anhydride (0.55 mL, 5.84 mmol, 6 eq.) and DMAP (11.9 mg, 90 µmol, 0.1 eq.). After shaking for 1 h at room temperature, the resin was washed with DMF (5 x 12 mL), DCM (3 x 12 mL) and Et_2O (3 x 12 mL) and dried *in vacuo*. The loading was measured *via* Fmoc-numbering (0.673 mmol/g).

Peptide synthesis The Fmoc-protected amino acids glycine (1.90 g, 2 eq.), alanine (0.68 g, 1 eq.), arginine(pbf) (5.45 g, 4 eq.), valine (0.75 g, 1 eq.), phenylalanine (0.85 g, 1 eq.), 6-amino-hexanoic acid (1.48 g, 2 eq.) and the preloaded HMBA-AM resin (1.22 g) were prepared for automated peptide synthesis (CEM Liberty). The activator HBTU (4.55 g, 0.2 M) and monohydrate HOBt (0.92 g, 0.1 M) in DMF (60 mL), the activator base DIPEA (10.5 mL) in NMP (19.5 mL), the deprotector piperidine 20% v/v (60 mL) in DMF (240 mL) and the solvents DMF (2.7 L) and DCM (83 mL) were added to the machine. The peptide was run under standard cycles and completed after 8.3 h. The resin was washed with DMF (3 x 12 mL), DCM (3 x 12 mL) and Et_2O (3 x 12 mL).

Boc protection To the resin in DMF (5 mL) was added Boc_2O (1.15 mL, 5.0 mmol, 10 eq.) at room temperature. After shaking for 16 h, *N*,*N*-diisopropylethylamine (DIPEA) (0.87 mL, 5.0 mmol, 10 eq.) was added. The reaction mixture was agitated for one hour, removed by filtration and the resin washed with DMF (3 x 20 mL), DCM (3 x 20 mL), MeOH (2 x 20 mL) and Et_2O (3 x 20 mL) and dried under reduced pressure.

Basic cleavage The peptide was cleaved from the resin with ice-cold NaOH (aq) (1 M, 3 mL) in 1,4-dioxane (9 mL) for 25 min. The resin was removed by filtration and wasing with MeCN (2 x 20 mL), H_2O/MeCN (1:1, 2 x 20 mL) and $H_2O_{dest.}$ (2 x 20 mL). The aqueous solution was neutralized to pH 6 with 1 M HCl (aq) and the solvent removed under reduced pressure. The crude mixture was purified by HPLC to give a white solid (297.6 mg, 39% overall).

HPLC Phenomenex Synergi 4u Hydro-RP 80A (100 x 21.2 µm, 4 micron), t_R = 10.0 min (H_2O/MeCN 55:45→5:95 over 12 min, 15 mL/min, 210 nm); **^{1}H NMR** (500 MHz, MeOD) δ_H 0.93 (dd, J = 4.9 Hz, J = 6.4 Hz, 6H), 1.17 (m, 2H), 1.31 (dt, ^3J = 7.5 Hz, ^3J = 15.0 Hz, 2H), 1.37 (m, 4H), 1.42 (s,

17H), 1.49 (m, 6H), 1.61 (dq, ^3J = 7.4 Hz, ^3J = 15.1 Hz, 3H), 1.75 (td, ^3J = 7.5 Hz, ^3J = 13.7 Hz, 1H), 2.05 (s, 4H), 2.15 (dd, 5H), 2.41 (m, 2H), 2.50 (s, 3H), 2.57 (s, 3H), 2.86 (dd, ^3J = 10.0 Hz, ^3J = 14.0 Hz, 1H), 2.96 (s, 2H), 3.01 (t, ^3J = 7.0 Hz, 2H), 3.08 (t, ^3J = 6.5 Hz, 3H), 3.14 (dd, ^3J = 5.1 Hz, ^3J = 14.0 Hz, 1H), 3.82 (m, 4H), 4.07 (m, 1H), 4.11 (m, 1H), 4.20 (q, ^3J = 7.5 Hz, 2H), 4.67 (dd, ^3J = 5.1 Hz, ^3J = 9.8 Hz, 1H), 7.18 (m, 4H), 7.24 (m, 16H); **^{1}H NMR** (500 MHz, DMSO-d$_6$) δ_H 0.80 (m, 6H), 1.03 (bs, 2H), 1.18 (m, 5H), 1.25 (m, 2H), 1.31 (s, 2H), 1.34 (s, 12H), 1.37 (s, 7H), 1.43 (m, 2H), 1.45 (m, 2H), 1.66 (bs, 2H), 1.84 (bs, 1H), 1.97 (bm, 8H), 2.26 (bs, 2H), 2.40 (s, 3H), 2.45 (s, 3H), 2.70 (t, ^3J = 11.9 Hz, 1H), 2.85 (d, ^3J = 5.3 Hz, 2H), 2.92 (bs, 4H), 2.97 (s, 1H), 3.00 (m, 2H), 3.15 (s, 2H), 3.67 (bm, 4H), 4.09 (bs, 1H), 4.12-4.20 (bs, 3H), 4.56 (bs, 1H), 6.72 (s, 1H), 7.14 (d, ^3J = 7.0 Hz, 10H), 7.22 (d, ^3J = 7.9 Hz, 10H), 7.65 (s, 1H), 7.86 (d, ^3J = 7.2 Hz, 1H), 8.01 (bm, 1H), 8.02 (bm, 1H), 8.06 (bm, 1H), 8.20 (m, 2H), 8.61 (s, 1H); **^{13}C NMR** (125 MHz, DMSO-d$_6$) δ_C 17.6, 17.8, 18.1, 19.0, 19.2, 25.0, 25.1, 25.9, 26.0, 28.3, 28.3, 28.9, 29.3, 30.6, 32.8, 35.1, 35.4, 37.2, 38.3, 40.4, 42.5, 48.6, 52.4, 53.6, 57.5, 69.2, 77.3, 86.3, 116.3, 124.3, 126.1, 126.3, 127.5, 127.9, 128.5, 129.3, 131.4, 134.3, 137.3, 138.1, 144.9, 155.6, 156.3, 157.4, 168.7, 170.8, 171.4, 171.6, 171.7, 171.8, 172.1, 172.5; **m/z** (EI+) calc. for [M+H]$^+$ = 1554.8065, found = 1554.8110.

2.4.2 1-(4′-Iodophenyl)-3,4-fulleropyrrolidine (5)[16]

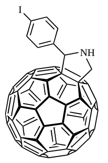

To a stirred solution of C_{60} fullerene (403.5 mg, 56.0 mmol, 1 eq.) and *para*-iodobenzaldehyde (42.0 mg, 56.0 mmol, 1 eq.) in anhydrous toluene/DMF 9:1 (200 mL, 3 mM) was added glycine (129.9 mg, 56.0 mmol, 1 eq.) under an atmosphere of argon. The solution was refluxed for 3.5 h and the solvent removed under reduced pressure. The crude product was purified by flash column chromatography on silica gel (CS_2→CS_2/toluene 1:1→toluene) to give unconsumed C_{60} (142.7 mg, 35%) and the monocyclo-adduct **5** (184.4 mg, 34%, 53% on recovered C_{60}). **R$_f$** 0.62 (toluene); **^{1}H NMR** (300 MHz, CDCl$_3$) δ_H 4.69 (bs, 1H), 4.86 (d, $^3J_{4a,4b}$ = 10.4 Hz, 1H), 5.09 (d, $^3J_{4b,4a}$ = 10.4 Hz, 1H), 5.74 (s, 1H), 7.59 (d, $^3J_{2',3'}$ = 7.3 Hz, 2H), 7.77 (d, $^3J_{3',2'}$ = 8.4 Hz, 2H); **^{13}C NMR** (75 MHz, CDCl$_3$) δ_C 61.6, 72.5, 80.9, 92.6, 129.1, 130.0, 136.1, 136.9, 137.4, 137.9, 139.8, 140.0, 140.1, 140.3, 141.7, 141.8, 142.0, 142.0 (2C), 142.1, 142.1, 142.2, 142.2, 142.3, 142.3, 142.4, 142.5, 142.7, 142.7, 142.7, 142.8, 143.1, 143.2, 144.4 (2C), 144.6, 144.7, 145.2, 145.3, 145.3, 145.3, 145.4 (2C), 145.4, 145.5, 145.5, 145.6, 145.7, 146.0 (2C), 146.1, 146.1, 146.2, 146.3, 146.3, 146.3, 146.4, 146.4, 146.6, 147.2 (2C), 152.4, 153.2 (2C), 153.7 (2C), 156.0; **m/z** (MALDI) calc. for [M] = 964.97, found = 964.80 (C_{60} standard m/z = 718.99).

2.4.3 *N*Boc-Ahx-Ahx-Phe-Val-Arg(pbf)-Gly-Ala-Gly-Glu(trt)-2-(4′-iodophenyl)-3,4-fulleropyrrolidine (6)

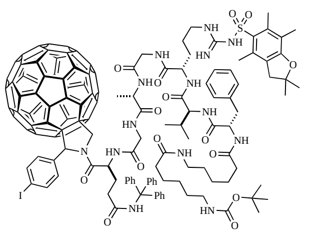

To a stirred solution of **5** (13.5 mg, 14.0 µmol, 1 eq.) in anhydrous $CHCl_3$ (4.3 mL, 3 mM) was added the bright yellow solution of preactivated peptide. This was prepared by adding DIPEA (3.7 µL, 21.0 µmol, 1.5 eq.) to a stirred solution of peptide **4** (21.8 mg, 14.0 µmol, 1 eq.) and HATU (8.0 mg, 21.0 µmol, 1.5 eq.) in anhydrous DMF (0.45 mL, 30 mM) and reacted at room temperature for 20 min until tlc analysis indicated complete conversion of starting material. The reaction mixture was stirred at room temperature for 1 h and 2.5 h at T=60 °C. The solvent was evaporated under reduced pressure. The crude product was purified by flash column chromatography on silica gel ($CHCl_3$→$CHCl_3$/MeOH 98:2→5:1) to give the peptide modified C_{60} **6** (14.9 mg, 43%, 81% on recovered **5**). **R$_f$** 0.22 ($CHCl_3$/MeOH 19:1); **IR** (film) ν_{max} 3290 (OH, NH), 2963-2857 (CH_3, CH_2, CH), 1696 (C=O amide), 1626 (C=O amide), 1519 (NH amide), 1447 (CH_2), 1367, 1248, 1167, 1107, 1091, 1006, 853, 752, 699 (C-I), 667; **^{1}H NMR** (500 MHz, DMSO-d$_6$) δ_H 0.82 (m, 6H), 1.04 (m, 2H), 1.13 (m, 5H), 1.22 (m, 3H), 1.23 (m, 2H), 1.28 (m, 2H), 1.29 (m, 2H), 1.36 (s, 9H), 1.38 (s, 7H), 1.40 (m, 2H), 1.46 (m, 2H), 1.64 (bs, 2H), 1.95-1.98 (m, 7H), 2.38 (s, 3H), 2.45 (s, 3H), 2.69 (m, 1H), 2.86 (m, 2H), 2.92 (bs, 3H), 3.02 (m, 2H), 3.71 (d, ^3J = 8.9 Hz, 2H), 3.84 (m, 2H), 4.22 (m, 2H), 4.57 (bs, 1H), 5.05 (bs, 3H), 5.70 (dd, ^3J = 12.1 Hz, ^3J = 28.7 Hz, 1H), 6.06 (bm, 1H), 6.38 (bs, 1H), 6.74 (t, ^3J = 5.3 Hz, 1H), 7.15 (d,

^3J = 7.6 Hz, 6H), 7.22 (m, 7H), 7.26 (m, 4H), 7.67 (s, 1H), 7.71 (d, ^3J = 12.0 Hz, 2H), 7.81 (m, 1H), 7.86 (d, ^3J = 8.0 Hz, 2H), 8.03 (bm, 2H), 8.18 (m, 1H), 8.39 (m, 1H), 8.72 (s, 1H); **^{13}C NMR** (125 MHz, DMSO-d$_6$) δ_C 17.7, 17.8, 18.3, 18.9, 25.0, 25.6, 25.8, 27.5, 28.0, 28.9, 29.0, 29.5, 30.2, 35.3, 35.2, 36.8 37.1, 38.0, 42.2, 39.6, 40.2, 41.7, 48.4, 50.6, 52.4, 53.4, 57.5, 58.4, 69.4, 69.8 77.4, 79.2, 86.3, 116.3, 124.4, 126.2, 126.4, 127.5 127.6, 128.0, 128.3, 128.6, 129.0, 129.9, 130.0, 131.5, 134.5, 137.4, 137.7, 138.1, 139.0, 139.4, 141.2, 141.5, 141.6, 142.1, 142.5, 143.8, 144.0, 144.1, 144.2, 144.7, 144.8, 144.8, 144.9, 145.0, 145.0, 145.3, 145.5, 145.6, 145.8, 145.8, 146.8, 154.5, 155.6, 156.1, 157.5, 162.4, 170.9, 171.4, 171.7, 171.9, 171.9, 172.2, **m/z** (MALDI) calc. for $[M+Na]^+$ = 2523.75, found = 2523.45 (C_{60} standard m/z = 720.38).

2.4.4 *N*-Ahx-Ahx-Phe-Val-Arg-Gly-Ala-Gly-Glu-2-(4'-iodophenyl)-3,4-fulleropyrrolidine (7)

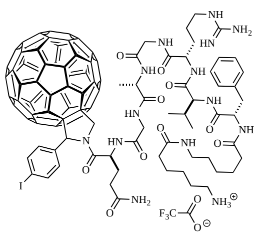

To fullerene coupled peptide **6** (8.8 mg, 3.5 µmol, 1 eq.) was added TFA/thioanisole (10:1, 1.87 mL, 1.9 mM) at room temperature. The reaction mixture was stirred for 40 min until tlc analysis indicated complete consumption of starting material. The solvent was removed under N_2 flow. The residual solid was washed with Et_2O (4 x 2 mL), sonicated for 2 min, centrifuged (2 min, 2 kG), and decanted after every wash. The brown solid was dried *in vacuo* to give the free amine functionalised fullerene **7** (6.1 mg, 91%). **IR** (ATR-FTIR on diamond) ν_{max} 3287 (OH, NH), 3065, 2961, 2936, 2868 (CH_3, CH_2, CH), 1778, 1643 (C=O amide), 1530 (NH amide), 1431 (CH_2), 1200, 1177, 1130, 1005, 833, 801, 719, 698 (C-I); **^{1}H NMR** (500 MHz, DMSO-d$_6$) δ_H 0.83 (bs, 6H), 1.05 (bs, 3H), 1.25 (m, 8H), 1.34 (s, 2H, H_{Ahx1}-5), 1.49 (m, 6H), 1.69 (bs, 2H), 2.00 (m, 6H), 2.76 (m, 3H), 2.93 (bs, 2H), 2.99 (m, 1H), 3.09 (bs, 2H), 3.79 (bm, 4H), 4.21-4.26 (m, 2H), 4.56 (bs, 1H), 5.18 (bs, 1H), 5.89 (m, 1H), 6.29 (m, 1H), 7.17 (bs, 1H), 7.24 (m, 4H), 7.41 (bs, 1H), 7.62 (bs, 2H), 7.71 (m, 2H), 7.87 (m, 2H), 8.06 (m, 3H), 8.22 (m, 1H), 8.37 (m, 1H); **^{13}C NMR** (125 MHz, DMSO-d$_6$) δ_C 17.7, 15.2, 18.0, 19.2, 24.8, 25.0, 25.5, 25.9, 26.9, 29.0, 29.0, 29.1, 30.8, 35.1, 35.2, 35.8, 37.1, 37.2, 38.4, 38.8, 41.9, 44.5, 48.2, 52.3, 53.6, 55.2, 57.4, 66.4, 67.4, 69.8, 89.4, 102.1, 125.9, 126.2, 128.0, 128.9, 129.2, 132.1, 137.6, 138.1, 141.2, 141.5, 141.6, 142.1, 142.5, 144.8, 145.0, 145.6, 145.6, 145.8, 156.6, 157.7, 170.9, 170.9, 171.4, 171.4, 171.5, 171.7, 172.2; **^{19}F NMR** (376 MHz, DMSO-d$_6$) δ_F -73.41; **m/z** (MALDI) calc. for $[M+H]^+$ = 1907.08, found = 1909.17 (C_{60} standard m/z = 718.95).

2.4.5 Attempted synthesis of CNT-XXFVRGAGQ-C_{60} (2)

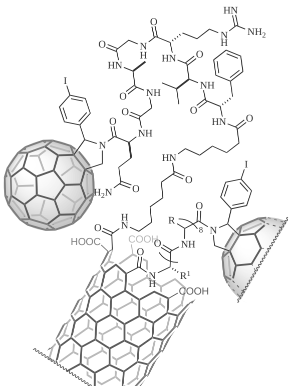

To oxidised CNT CNTCOOH (**1**) (2.7 mg, 1 eq.) was added $SOCl_2$ (0.5 mL/mg) under an atmosphere of argon. The suspension was refluxed for 15 h under vigorous stirring. The solvent was removed under reduced pressure. The solid was washed with anhydrous THF (3 x 4 mL), centrifuged (20 min, 7197 G), decanted the yellow solution and dried the black powder *in vacuo*.

To a vigorously stirred suspension of this preactivated CNT-COCl (3.2 mg, 1 eq.) in anhydrous $CHCl_3$ (1 ml/mg) were added the peptide functionalised fulleropyrrolidine **7** (1.7 mg, 0.5 mass eq.) and DIPEA (0.3 µL, 2 eq. to amine) under an atmosphere of argon at 70 °C and stirred for 87 h. The solvent was removed under reduced pressure. The crude mixture was washed with DMSO (3 x 3 mL), sonicated for 10 min, centrifuged (15 min, 2 kG), decanted. The procedure was repeated with H_2O (2 mL), MeCN (2 x 2 mL), DCM (2 mL) and dried *in vacuo* to give CNT-XXFVRGAGQ-C_{60} construct **2** as black solid (4.1 mg). **ATR-FTIR** ν_{max} 2959, 2928, 2854 (CH, CH_2), 2671, 2347 (OH, NH), 1689 (C=O amide), 1597 (C=C), 1462, 1377, 1265, 1223, 1126, 824; **Raman** A_D/A_G = 0.1409 (+33%); $\tilde{\nu}_{max}$ [cm^{-1}] 151, 163, 192, 214, 242, 270, 286, 301, 309, 335, 345, 678, 704, 775, 901, 1052, 1315, 1577, 1713, 1906, 2607; **HRTEM** 11 ends: 10 (91%) open thereof 7 (70%) functionalised and 4 (40%) filled tubes; **EDX** C, Si, O, Cl, S, I, Fe detected; **XRF** S, Cl, Ar, Ca, I, Fe, Ni detected; **XPS** I, O, N, C present; 1 x I per 321 carbon

and 12 nitrogen; every 13th COOH functionalised; **TGA** (in air) $T_{combust.}$ = 278.9, 335(sh), 508.1(sh), 623.1 °C, 1.21% residue.

2.5 Attempted Synthesis of Hybrid 3: XXFVIGAGQ functionalised C_{60} coupled to CNT-COOH

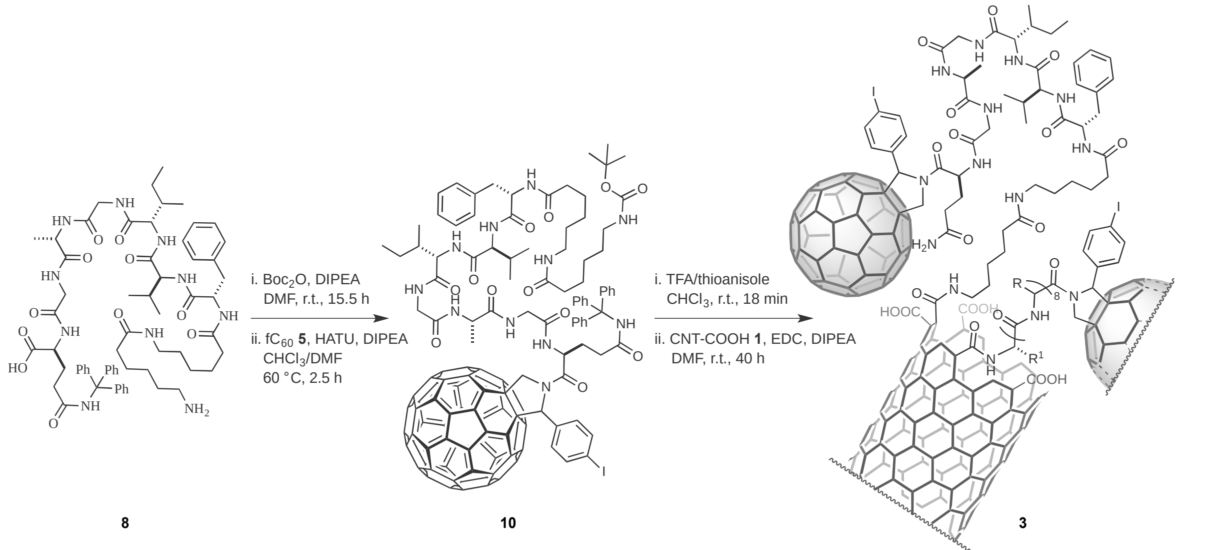

8 10 3

***Supplementary Scheme S2**: Synthesis scheme for CNT-C_{60} construct **3** based on peptide **8,** coupled to the fullerene cork to give **10**.*

2.5.1 NH_2-Ahx-Ahx-Phe-Val-Ile-Gly-Ala-Gly-Gln(trt)-OH (8)[15]

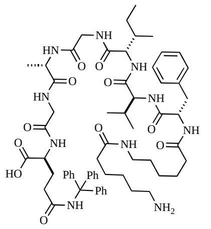

Peptide synthesis. The Fmoc-protected amino acids glycine (0.65 g, 2 eq.), alanine (0.37 g, 1 eq.), isoleucine (0.42 g, 1 eq.), valine (0.41 g, 1 eq.), phenylalanine (0.46 g, 1 eq.), 6-aminohexanoic acid (0.78 g, 2 eq.) and glutamine(trt) preloaded 2-chlorotrityl resin (0.368 g, 0.68 mmol/g) were prepared for automated peptide synthesis (CEM Liberty). The activator HBTU (1.897 g, 0.2 M) in DMF (25 mL), activator base DIPEA (5.2 mL) in NMP (9.8 mL), deprotector piperidine 20% v/v (40 mL) in DMF (160 mL) and the solvents DMF (1.6 L) and DCM (83 mL) were added to the machine. The peptide was run with standard cycles and was completed after 5.5 h. The resin was washed by filtration with DMF (3 x 10 mL), DCM (3 x 10 mL) and Et_2O (2 x 10 mL) and dried *in vacuo*.

Acidic cleavage The peptide was cleaved from the resin with a mixture of HFIP/DCM 1:4 (10 mL, 27 mL/g) by agitation for 10 min[17]. The resin was filtered and washed with DCM (2 x 5 mL), MeCN (2 x 8 mL), MeCN/H_2O 1:1 (10 mL), MeOH (10 mL), DCM (2 x 8 mL), Et_2O (10 mL) and the solvent removed under reduced pressure. The crude peptide was first purified by trituration with cold Et_2O/hexane 1:1 (40 mL), centrifugation (15 min, 7197 G) and decantation to give crude peptide **8** (113.0 mg, 26%). 30 mg of the crude mixture was purified by HPLC to give a white solid (19.0 mg, 4%).

R_f 0.0 ($CHCl_3$/MeOH 19:1+1 drop FA); **HPLC** Phenomenex Synergi 4u Hydro-RP 80A (100 x 21.2 µm, 4 micron), t_R = 15.2 min (H_2O/MeCN 95:5→5:95, 24 min, 1 mL/min, 210 nm); **IR** (film) ν_{max} 3726-3629, 3289 (OH, NH), 2938 (CH_3, CH_2, CH), 1626 (C=O amide), 1541 (NH amide), 1457 (CH_2), 1054, 1033, 1012, 667, 650, 625; **^{1}H NMR** (500 MHz, DMSO-d_6) δ_H 0.82 (m, 12H), 1.08 (m, 3H), 1.22 (d, ^3J = 6.6 Hz, 5H), 1.28 (m, 3H), 1.34 (m, 2H), 1.47 (m, 5H), 1.72 (bs, 2H), 1.84 (bs, 1H), 2.01 (m, 5H), 2.25 (bs, 2H), 2.75 (m, 3H), 2.94 (bs, 2H), 3.01 (dd, ^2J = 3.2 Hz, ^3J = 13.5 Hz, 1H), 3.71 (m, 4H), 4.00 (bs, 1H), 4.10 (t, ^3J = 7.7 Hz, 1H), 4.23 (dd, ^3J = 7.2 Hz, ^3J = 14.7 Hz, 2H), 4.53 (td, ^3J = 4.0 Hz, ^3J = 10.2 Hz, 1H), 7.18 (m, 11H), 7.24 (m, 10H), 7.49 (d, ^3J = 4.5 Hz, 1H), 7.75 (bs, 1H), 8.01 (d, ^3J = 6.4 Hz, 1H), 8.11 (d, ^3J = 6.7 Hz, 1H), 8.20 (d, ^3J = 8.2 Hz, 1H), 8.26 (m, 2H), 8.44 (m, 1H), 8.76 (s, 1H); **^{13}C NMR** (125 MHz, DMSO-d_6) δ_C 11.0, 15.3, 17.9, 18.2, 19.3, 24.6, 24.8, 25.0, 25.6, 25.9, 26.8, 27.0, 28.9, 29.2, 30.7, 33.3, 35.1, 36.2, 37.2, 38.6, 40.4, 42.0, 42.4, 48.4, 53.4, 54.0, 57.6, 57.8, 69.2,

126.1, 126.3, 127.5, 128.0, 128.6, 129.2, 138.2, 145.0, 167.9, 168.6, 171.0, 171.4, 171.4, 171.8, 172.0, 172.2, 172.5, 174.0; **m/z** (ESI+) calc. for $[M]^+$ = 1159.6538, found = 1159.6550.

2.5.2 NHBoc-Ahx-Ahx-Phe-Val-Ile-Gly-Ala-Gly-Gln(trt)-OH (9)

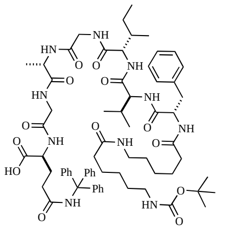

To a stirred dispersion of **8** (83.1 mg, 71.7 µmol, 1 eq.) in anhydrous DMF (9 mL, 8 mM) was added Boc_2O (0.21 mL, 0.91 mmol, 13 eq.) at room temperature. After stirring for 14.5 h DIPEA (78.9 µL, 0.45 mmol, 6 eq.) was added. The reaction mixture was stirred for one hour until tlc indicated complete conversion of starting material and the solvent removed under reduced pressure. The crude peptide was first purified by trituration with cold Et_2O/hexane 1:1 (2 x 30 mL), centrifugation (10 min, 7197 G) and decantation to give the crude mixture (87.7 mg, 97%) which was further purified by HPLC to give peptide **9** as a white solid (40.7 mg, 45%). **R_f** 0.72 ($CHCl_3$/MeOH/FA 40:10:1); **HPLC** Phenomenex Synergi 4u Hydro-RP 80A (100 x 21.2 µm, 4 micron), t_R = 18.2 min (H_2O/MeCN 95:5→5:95, 24 min, 1 mL/min, 210 nm); t_R = 19.2 min is an isomeric form (2.8 mg, 3%); **IR** (film) ν_{max} 3725-3629, 3283 (OH, NH), 2966-2867 (CH_3, CH_2, CH), 1627 (C=O amide), 1523 (NH amide), 1456 (CH_2), 1396, 1366, 1252, 1170, 1055, 1033, 1013, 669, 649, 618; **^{1}H NMR** (500 MHz, DMSO-d_6) δ_H 0.82 (m, 12H), 1.07 (m, 3H), 1.19 (m, 6H), 1.27 (m, 2H), 1.33 (m, 4H), 1.36 (s, 9H), 1.44 (m, 3H), 1.72 (m, 2H), 1.89 (m, 1H), 1.98 (m, 5H), 2.30 (m, 2H), 2.73 (dd, ^3J = 10.4 Hz, ^3J = 13.8 Hz, 1H), 2.87 (dd, ^3J = 6.8 Hz, ^3J = 12.9 Hz, 2H), 2.93 (dd, ^3J = 6.8 Hz, ^3J = 12.9 Hz, 2H), 2.99 (dd, ^2J = 3.9 Hz, ^3J = 13.8 Hz, 1H), 3.70 (dd, ^3J = 5.3 Hz, ^3J = 11.8 Hz, 4H), 4.11 (m, 1H), 4.13 (m, 1H), 4.21 (m, 1H), 4.25 (m, 1H), 4.58 (td, ^3J = 4.1 Hz, ^3J = 10.1 Hz, 1H), 6.75 (t, ^3J = 5.4 Hz, 1H), 7.18 (m, 11H), 7.25 (m, 10H), 7.68 (t, ^3J = 5.4 Hz, 1H), 7.88 (d, ^3J = 7.2 Hz, 1H), 7.95 (m, 3H), 8.05 (d, ^3J = 8.4 Hz, 1H), 8.21 (m, 2H), 8.64 (s, 1H); **^{13}C NMR** (125 MHz, DMSO-d_6) δ_C 11.0, 15.3, 18.0, 18.1, 19.2, 24.5, 25.0, 25.1, 25.9, 26.0, 27.6, 28.3, 28.9, 29.3, 30.6, 32.7, 35.1, 35.4, 36.4, 37.3, 38.3, 39.6, 40.4, 41.9, 41.9, 48.4, 51.9, 53.7, 57.2, 57.6, 69.2, 77.3, 126.1, 126.3, 127.5, 127.9, 128.5, 129.2, 138.1, 144.9, 155.6, 168.5, 168.6, 170.9, 171.2, 171.4, 171.4, 171.8, 172.1, 172.5, 173.4; **m/z** (ESI+) calc. for $[M]^+$ = 1259.7059, found = 1259.7075.

2.5.3 *N*Boc-Ahx-Ahx-Phe-Val-Ile-Gly-Ala-Gly-Glu(trt)-2-(4′-iodophenyl)-3,4-fulleropyrrolidine (10)

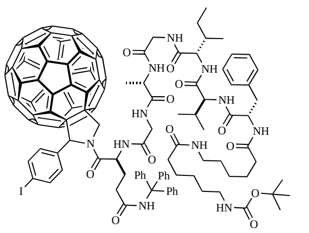

To a stirred solution of fulleropyrrolidine **5** (14.0 mg, 14.5 µmol, 1 eq.) in anhydrous $CHCl_3$ (4.2 mL, 3 mM) was added a bright yellow solution of preactivated peptide. This was prepared by adding DIPEA (3.79 µL, 21.8 µmol, 1.5 eq.) to a stirred solution of peptide **9** (18.3 mg, 14.5 µmol, 1 eq.) and HATU (16.6 mg, 43.6 µmol, 3 eq.) in anhydrous DMF (0.4 mL, 30 mM) reacted at room temperature for 30 min until tlc analysis indicated complete conversion of starting material. The reaction mixture was stirred for 21 h at T=60°C, diluted with $CHCl_3$ (20 mL) and H_2O (20 mL) and extracted with $CHCl_3$ (3 x 10 mL). The combined organic layers were dried over $MgSO_4$, filtered and the solvent was removed under reduced pressure. The crude product was purified by flash column chromatography on silica gel ($CHCl_3$→$CHCl_3$/MeOH 49:1→5:1) to give the coupled product **10** (7.9 mg, 25%, 33% on recovered **5**). **R_f** 0.50 ($CHCl_3$/MeOH 9:1); **^{1}H NMR** (500 MHz, DMSO-d_6) δ_H 0.82 (m, 12H), 1.06 (m, 3H), 1.21 (m, 11H), 1.32 (m, 5H), 1.36 (s, 6H), 1.45 (m, 3H), 1.68 (m, 1H), 1.98 (m, 5H), 2.72 (m, 1H), 2.87 (dd, ^3J = 6.8 Hz, ^3J = 13.2 Hz, 2H), 2.94 (m, 2H), 2.98 (dd, ^2J = 3.5 Hz, ^3J = 14.0 Hz, 1H), 3.66 (dd, ^3J = 4.1 Hz, ^3J = 16.8 Hz, 1H), 3.71 (m, 1H), 3.76 (d, ^3J = 16.9 Hz, 1H), 3.82 (d, ^3J = 22.9 Hz, 1H), 4.12 (m, 1H), 4.22 (m, 2H), 4.30 (m, 1H), 4.57 (bs, 1H), 5.08 (m, 1H), 5.67 (m, 1H), 6.08 (m, 1H), 6.75 (t, ^3J = 5.1 Hz, 1H), 7.13 (m, 8H), 7.21 (m, 7H), 7.28 (d, ^3J = 7.7 Hz), 7.66 (d, ^3J = 4.4 Hz, 1H), 7.72 (dd, ^3J = 9.7 Hz, ^3J = 17.9 Hz, 1H), 7.85 (m, 3H), 8.00 (m, 1H), 8.18 (m, 1H), 8.26 (bs, 1H), 8.37 (bs, 1H), 8.69 (s, 1H); **^{13}C NMR** (125 MHz, DMSO-d_6) δ_C 11.1, 15.3, 18.0, 18.1, 19.2, 24.4, 25.0, 25.1, 25.9, 26.0, 28.3, 28.9, 29.0, 29.3, 30.5, 31.3, 32.0, 35.1, 35.4, 36.4, 37.2, 38.3, 39.8, 41.9, 41.9, 48.4, 48.6, 53.4/6, 57.0/1, 57.5, 69.3, 69.8, 77.3, 78.7, 79.0, 79.2, 79.2, 126.1, 126.3, 127.4, 127.5, 127.9, 128.6, 129.2, 130.0, 135.5, 136.2, 137.7, 138.1, 139.1, 139.4, 139.5, 141.1, 141.4, 141.6, 141.7, 142.1,

142.5, 143.8, 144.0, 144.1, 144.8, 144.9, 145.0, 145.3, 145.5, 145.6, 145.8, 146.8, 146.9, 147.4, 152.3, 155.6, 169.1, 170.9, 171.3, 171.8, 172.0, 172.5; **m/z** (MALDI) calc. for [M+Na] = 2228.65, found = 2228.99 (C_{60} standard m/z = 718.73).

4.5.4 *N*H_2-Ahx-Ahx-Phe-Val-Ile-Gly-Ala-Gly-Glu-2-(4'-iodophenyl)-3,4-fulleropyrrolidine (11)

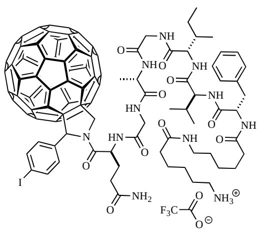

To a stirred solution of **10** (8.1 mg, 3.6 µmol, 1 eq.) in anhydrous $CHCl_3$ (3.6 mL, 1 mM) was added a prepared solution of TFA/thioanisole 10:1 (3.96 mL, 1 mM) at room temperature. The reaction mixture was stirred for 18 min until tlc analysis indicated complete consumption of starting material. The solvent was removed under reduced pressure. The crude product was purified by trituration with Et_2O (3 x 9 mL), centrifugation (10 min, 7197 G) and decantation and dried *in vacuo* to give the deprotected product **11** (5.7 mg, 84%). **IR** (film) ν_{max} 3710-3629, 3287 (OH, NH), 2967-2826 (CH_3, CH_2, CH), 2075, 1629 (C=O amide), 1540 (NH amide), 1456 (CH_2), 1436„ 1346, 1204, 1135, 1055, 1033, 1010, 820, 801, 751, 721, 668; **Raman** $\tilde{\nu}_{max}$ [cm^{-1}] 268, 492, 1558 (b), 2306 (b); **^{1}H NMR** (500 MHz, DMSO-d_6) δ_H 0.82 (m, 12H), 1.06 (m, 3H), 1.23 (m, 7H), 1.34 (m, 3H), 1.49 (m, 5H), 1.70 (m, 1H), 2.00 (m, 5H), 2.74 (m, 3H), 2.96 (m, 3H), 3.74 (m, 4H), 4.15 (m, 1H), 4.24 (m, 2H), 4.57 (m, 1H), 5.19 (m, 1H), 5.87 (dd, ^3J = 12.8 Hz, ^3J = 59.2 Hz, 1H), 6.32 (dd, ^3J = 10.1 Hz, ^3J = 83.5 Hz, 1H), 6.87 (s, 1H), 7.16 (m, 1H), 7.21 (m, 4H), 7.32 (m, 1H), 7.64 (bs, 2H), 7.70 (bs, 1H), 7.76 (m, 2H), 7.87 (m, 3H), 8.03 (m, 2H), 8.23 (m, 1H); **^{13}C NMR** (125 MHz, DMSO-d_6) δ_C 11.1, 15.3, 18.0, 18.1, 19.2, 24.4, 24.8, 25.0, 25.5, 25.9, 26.9, 29.0, 30.4, 30.6, 35.1, 36.5, 37.3, 38.3, 38.8, 41.9, 48.6, 50.4, 53.6, 57.0, 57.5, 69.9, 124.9, 126.1, 127.9, 129.2, 130.0, 135.6, 136.3, 137.7, 138.1, 139.1, 139.4, 139.6, 141.2, 141.5, 141.6, 141.6, 141.6, 141.7, 142.0, 142.1, 142.5, 142.6, 143.8, 144.0, 144.1, 144.2, 144.7, 144.8, 144.9, 145.0, 145.0, 145.3, 145.5, 145.6, 145.8, 145.8, 146.8, 146.9, 157.8, 168.6, 169.1, 170.5, 170.8, 171.2, 171.4, 171.6, 172.0, 172.5, 173.9; **m/z** (MALDI) calc. for $[M+H]^+$ = 1864.51, found = 1865.15 (C_{60} standard m/z = 718.60).

4.5.5 Attempted Synthesis of CNT-XXFVIGAGQ-C_{60} (3)

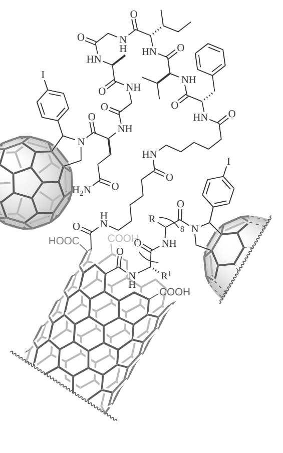

To a suspension of oxidised CNT **1** (5 mg, 1 eq.) in anhydrous DMF (2.5 mL, 0.5 ml/mg) were added EDC (10 mg, 2 mass eq.) and DIPEA (9.1 µL, 2 eq. to amine) at room temperature under an atmosphere of argon. The mixture was sonicated for 1 h. Peptide functionalised C_{60} (**11**, 1.9 mg, 0.5 mass eq.) was added to the mixture, sonicated for 1 h and stirred vigorously for 39 h at room temperature. The crude mixture was washed with H_2O (3 x 11 mL), sonicated for 30 min, centrifuged (20 min, [illegible]197 G), decanted. The washing procedure was repeated with DMF (3 x 11 mL), MeCN (3 x 11 mL), Et_2O (3 x 11 mL) and the product dried *in vacuo* to give CNT-XXFVIGAGQ-C_{60} construct **3** as a black solid (5.8 mg). **ATR-FTIR** ν_{max} 2590, 1694 (shoulder, C=O amide), 1607 (C=C), 1505, 1354, 1267, 1123; **Raman** A_D/A_G = 0.1210 (+1%); $\tilde{\nu}_{max}$ [cm^{-1}] 151, 162, 189, 214, 244, 284, 301, 309, 334, 345, 678, 773, 896, 1051, 1318, 1574, 1715, 1901, 2605; **HRTEM** 35 ends: 12 closed, 13 (37%) open thereof 11 (85%) functionalised and 5 (38%) filled tubes; **EDX** C, O, I, Fe, Ca, Si, S, Cl detected; **XRF** Cl, Ar, Ca, I, Ba, Cr, Fe, Ni detected; **XPS** I, O, N, C present; 1 x I per 613 carbon and 11 nitrogen; every 16th COOH functionalised; **TGA** (in air) $T_{combust.}$ = 329.9, 596.9, 629(sh) °C, 1.64% residue.

3. The bounds of the 3D contrast transfer function.

To explain why WDD reconstruction offers a true optical sectioning effect rather than a Fresnel propagated version of the exit wave, we start with equation 2 of the main text. Consider a thin sample that is located a distance, z, from the focal plane of the microscope along the electron beam direction. To make this effective defocus explicit in the mathematics, we write the aperture function, A, as the product of an amplitude term, H, that has a value of unity inside the aperture and zero outside, and a phase term reflecting the usual defocus aberration term, or equivalently the *Fresnel* propagation term,

$$A(\mathbf{K_f}) = H(\mathbf{K_f})\exp\left(i\pi\lambda z \mathrm{K_f}^2\right) \qquad (11)$$

Substitution into equation 2 of the main text gives, after some simplification,

$$G(\mathbf{K_f}, \mathbf{Q_p}) = \left\{H(\mathbf{K_f})H(\mathbf{K_f} + \mathbf{Q_p})\exp\left[-i\pi\lambda z\left(\mathrm{Q_p}^2 + 2\mathbf{K_f}\cdot\mathbf{Q_p}\right)\right]\right\} \otimes_{\mathbf{K_f}} \Psi(\mathbf{K_f})\Psi^*(\mathbf{K_f} - \mathbf{Q_p}) \qquad (12)$$

The function on the left of the convolution in equation 2 now represents the overlap between two discs with a phase that varies linearly with position, $\boldsymbol{K_f}$, within the overlap region. In the weak phase object approximation, equation 12 can be written as

$$\begin{aligned} G(\mathbf{K_f}, \mathbf{Q_p}) = {} & |H(\mathbf{K_f})|^2\delta(\mathbf{Q_p}) + \left\{H(\mathbf{K_f})H(\mathbf{K_f} + \mathbf{Q_p})\exp\left[i\pi\lambda z\left(-\mathrm{Q_p}^2 - 2\mathbf{K_f}\cdot\mathbf{Q_p}\right)\right]\right\}\Psi'^*(-\mathbf{Q_p}) \\ & + \left\{H(\mathbf{K_f})H(\mathbf{K_f} - \mathbf{Q_p})\exp\left[i\pi\lambda z\left(\mathrm{Q_p}^2 - 2\mathbf{K_f}\cdot\mathbf{Q_p}\right)\right]\right\}\Psi'(\mathbf{Q_p}) \end{aligned}$$

(13)

The phase variation in the overlap region due to aberrations can be seen clearly in Supplementary Fig.2. In the single side-band ptychography method used previously [18], one side of the disc double-overlap region is simply integrated in $\mathbf{K_f}$ to get the overall amplitude and phase of the Fourier component of the reconstructed phase image at the spatial frequency, $\mathbf{Q_p}$. If the phase is not constant, for example if the object is out of focus, then the integration over the phase variation across the overlap region will result in a lower overall magnitude of that Fourier component and a reduced contribution to the reconstruction. Objects in focus, where there will be no phase variation, will contribute more strongly.

In the WDD approach, the deconvolution step uses a kernel that corresponds to a disc overlap region, and an intentional defocus term can be used in the kernel resulting in a phase variation across the disc overlap region. Because the deconvolution step aims to reduce a disc overlap feature in $G(\mathbf{K_f}, \mathbf{Q_p})$ to a single point after deconvolution, it can be regarded as having an implicit summation over the disc overlap region. If the defocus set in the kernel matches that in the experimental data, the phase variation will be corrected and the sum over the disc overlap region will give a strong contribution. If there is a mismatch between the experimental and deconvolution defocus, there will be a resultant phase variation and the strength of the contribution will be reduced.

Under the WPO approximation, the 2D transfer function with zero aberrations is given by the integration over the disc-overlap region[19]. To extend this approach to 3D in the case of WDD, the strength of the transfer for a spatial frequency, $\mathbf{Q_p}$, for an object a distance, z, from the defocus used for the deconvolution, can be computed by integrating over the convolution kernel in equation 13,

$$T(\mathbf{Q_p}, z) = \int H(\mathbf{K_f})H(\mathbf{K_f} + \mathbf{Q_p})\exp\left[i\pi\lambda z\left(-\mathrm{Q_p}^2 - 2\mathbf{K_f}\cdot\mathbf{Q_p}\right)\right]\, d\mathbf{K_f} \qquad (14)$$

To express the transfer function entirely in reciprocal space we take the Fourier transform with respect to *z*, which can be written explicitly as

$$T(\mathbf{Q_p}, z^*) = \iint H(\mathbf{K_f})H(\mathbf{K_f} + \mathbf{Q_p})\exp[i2\pi z z^*]\exp\left[i\pi\lambda z\left(-\mathrm{Q_p}^2 - 2\mathbf{K_f}\cdot\mathbf{Q_p}\right)\right]\, dz\, d\mathbf{K_f} \qquad (15)$$

where z^* is the conjugate reciprocal space variable to *z*. The *z*-integral can be performed first, and can be written

$$\int \exp\left[i2\pi z\left(z^* + \tfrac{1}{2}\lambda\left[-\mathrm{Q_p}^2 - 2\mathbf{K_f}\cdot\mathbf{Q_p}\right]\right)\right]\, dz = \delta\left(z^* + \tfrac{1}{2}\lambda\left[-\mathrm{Q_p}^2 - 2\mathbf{K_f}\cdot\mathbf{Q_p}\right]\right) \qquad (16)$$

where δ is the Dirac delta-function.

Equation 16 allows us to explore the bounds of the 3D transfer function. Remembering that in the integration over $\mathbf{K_f}$ in equation 15, the domain of integration of $\mathbf{K_f}$ is bounded by the functions, *H*, leading to an area that is the overlap between a disc at **0** and a disc at $-\mathbf{Q_p}$, the maximum and minimum values of z^* allowed by the Dirac delta-function can be found for $\mathbf{K_f}$ having a magnitude corresponding to the aperture radius, α, and a direction parallel to $-\mathbf{Q_p}$, or a magnitude corresponding to $Q_p - \alpha$ in the same direction (see Supplementary Fig. 12). The first condition can be substituted into equation 16 to give

$$\delta\left(z^* + \frac{1}{2}\lambda\left[-Q_p{}^2 + 2\alpha Q_p\right]\right)$$

and the second condition gives

$$\delta\left(z^* + \frac{1}{2}\lambda\left[Q_p{}^2 - 2\alpha Q_p\right]\right)$$

The values of z^* allowed by these two delta functions are two parabolas, and 3D transfer is provided between these two bounds. The bounds are identical to those seen for annular dark-field STEM optical sectioning[20], and are shown in Supplementary Fig. 13. The similar bounds for transfer have been discussed for tilt-series reconstruction in HRTEM[21] which, by reciprocity, is equivalent to ptychography. The use of the entire disc overlap function in ptychography plays a similar role to the integration over the detector function in ADF STEM. For the single axial illumination used in HRTEM or exit-wave reconstruction, the 3D transfer only occurs on the truncated surface of a sphere. This difference in transfer function explains why ptychography shows a real optical sectioning effect, rejecting out of plane objects, that is not seen by performing *Fresnel* propagation of a reconstructed exit-wave.

Supplementary References

1. Press, W. H., Teukolsky, S. A., Vetterling, W. T. & P., F. B. *Numerical recipes in C++*. (Cambridge University Press, 2003).
2. Krivanek, O. L., Dellby, N. & Lupini, A. R. Towards sub-Å electron beams. *Ultramicroscopy* **78,** 1–11 (1999).
3. Bernier, P. *et al.* Large-scale production of single-walled carbon nanotubes by the electric-arc technique. *Nature* **388,** 756–758 (1997).
4. Lu, J. *et al.* Fabrication of Ordered Catalytically Active Nanoparticles Derived from Block Copolymer Micelle Templates for Controllable Synthesis of Single-Walled Carbon Nanotubes. *J. Phys. Chem. B* **110,** 6655–6660 (2006).
5. Tobias, G., Shao, L., Salzmann, C. G., Huh, Y. & Green, M. L. H. Purification and Opening of Carbon Nanotubes Using Steam. *J. Phys. Chem. B* **110,** 22318–22322 (2006).
6. Ballesteros, B. *et al.* Steam Purification for the Removal of Graphitic Shells Coating Catalytic Particles and the Shortening of Single-Walled Carbon Nanotubes. *Small* **4,** 1501–1506 (2008).
7. Still, W. C., Michael, K. & Mitra, A. Rapid Chromatographic Technique for Preparative Separations with Moderate Rsolution. *J. Org. Chem.* **43,** 2923–2925 (1978).
8. Tchoul, M. N., Ford, W. T., Lolli, G., Resasco, D. E. & Arepalli, S. Effect of Mild Nitric Acid Oxidation on Dispersability, Size, and Structure of Single-Walled Carbon Nanotubes. *Chem. Mater.* **19,** 5765–5772 (2007).
9. Shao, L. *et al.* Removal of amorphous carbon for the efficient sidewall functionalisation of single-walled carbon nanotubes. *Chem. Commun.* **1,** 5090–5092 (2007).
10. Shao, L., Lin, T.-W., Tobias, G. & Green, M. L. H. A simple method for the containment and purification of filled open-ended single wall carbon nanotubes using C60 molecules. *Chem. Commun.* **18,** 2164-2166 (2008).
11. Ziegler, K. J. *et al.* Controlled Oxidative Cutting of Single-Walled Carbon Nanotubes. *J. Am. Chem. Soc.* **127,** 1541–1547 (2005).
12. Hamon, M. A. *et al.* Dissolution of Single-Walled Carbon Nanotubes. *Adv. Mater.* **11,** 834–840 (1999).
13. Chen, R. J., Zhang, Y., Wang, D. & Dai, H. Noncovalent Sidewall Functionalization of Single-Walled Carbon Nanotubes for Protein Immobilization. *J. Am. Chem. Soc.* **123,** 3838–3839 (2001).
14. He, Z. & Zhou, J. Probing carbon nanotube–amino acid interactions in aqueous solution with molecular dynamics simulations. *Carbon N. Y.* **78,** 500–509 (2014).
15. Chan, W. C. & White, P. D. *Fmoc Solid Phase Synthesis – A Practical Approach*. (Oxford University Press, 2000).
16. Hong, S. Y. *et al.* Filled and glycosylated carbon nanotubes for in vivo radioemitter localization and imaging. *Nat. Mater.* **9,** 485–490 (2010).
17. Bollhagen, R., Schmiedberger, M., Barlos, K. & Grell, E. A new reagent for the cleavage of fully protected peptides synthesised on 2-chlorotrityl chloride resin. *J. Chem. Soc. Chem. Commun.* **22**, 2559–2560 (1994).
18. Pennycook, T. J. *et al.* Efficient phase contrast imaging in STEM using a pixelated detector. Part 1: Experimental demonstration at atomic resolution. *Ultramicroscopy* **151,** 160–167

(2015).

19. Yang, H., Pennycook, T. J. & Nellist, P. D. Efficient phase contrast imaging in STEM using a pixelated detector. Part II: Optimisation of imaging conditions. *Ultramicroscopy* **151,** 232–239 (2015).

20. Cosgriff, E. C. *et al.* in *Advances in Imaging and Electron Physics, Vol 162* (ed. Hawkes, P. W.) **162,** 45–76 (2010).

21. Kirkland, A. I., Saxton, W. O., Chau, K.-L., Tsuno, K. & Kawasaki, M. Super-resolution by aperture synthesis: tilt series reconstruction in CTEM. *Ultramicroscopy* **57,** 355–374 (1995).
